# Comparison of multi-lineage differentiation of hiPSCs reveals novel miRNAs that regulate lineage specification

**DOI:** 10.1038/s41598-018-27719-0

**Published:** 2018-06-25

**Authors:** Lu Li, Kai-Kei Miu, Shen Gu, Hoi-Hung Cheung, Wai-Yee Chan

**Affiliations:** 1 CUHK-CAS GIBH Joint Research Laboratory on Stem Cell and Regenerative Medicine, School of Biomedical Sciences, the Chinese University of Hong Kong, Shatin, N.T. Hong Kong SAR; 2 Present Address: School of Pharmacy, University of Southern California, Los Angeles, CA USA; 3 Present Address: M&H Genetics/Baylor Genetics Laboratories, Baylor College of Medicine, Houston, TX USA

## Abstract

MicroRNAs (miRNAs) are known to be crucial players in governing the differentiation of human induced pluripotent stem cells (hiPSCs). Despite their utter importance, identifying key lineage specifiers among the myriads of expressed miRNAs remains challenging. We believe that the current practice in mining miRNA specifiers via delineating dynamic fold-changes only is inadequate. Our study, therefore, provides evidence to pronounce “lineage specificity” as another important attribute to qualify for these lineage specifiers. Adopted hiPSCs were differentiated into representative lineages (hepatic, nephric and neuronal) over all three germ layers whilst the depicted miRNA expression changes compiled into an integrated atlas. We demonstrated inter-lineage analysis shall aid in the identification of key miRNAs with lineage-specificity, while these shortlisted candidates were collectively known as “lineage-specific miRNAs”. Subsequently, we followed through the fold-changes along differentiation via computational analysis to identify *miR-192* and *miR-372-3p*, respectively, as representative candidate key miRNAs for the hepatic and nephric lineages. Indeed, functional characterization validated that *miR-192* and *miR-372-3p* regulate lineage differentiation via modulation of the expressions of lineage-specific genes. In summary, our presented miRNA atlas is a resourceful ore for the mining of key miRNAs responsible for lineage specification.

## Introduction

Extensive evidence has shown that human pluripotent stem cells (hPSCs) can give rise to all somatic cell types in an adult human^1–4^. Understanding the mechanisms by which hPSCs differentiate during early embryogenesis is crucial to understanding human development. Moreover, a comprehensive understanding of the mechanisms underlying hPSC differentiation is required for their application in disease studies, where intentionally differentiated functional cells derived from hPSCs have been widely used for disease modeling and replacement therapy^5–7^.

A previous in-depth analysis of the transcriptome reported a temporal change in microRNA (miRNA) expression levels during hPSC differentiation^8^, suggesting that the expression dynamics of miRNAs are associated with hPSC differentiation^9^. In addition, the identification of miRNAs regulating lineage specification such as *miR-375*, which regulates pancreatic islet formation, further confirms that miRNAs can serve as cell fate determinants^8,10–15^. However, identification of miRNAs regulating lineage specification, hereafter referred to as key miRNAs in this manuscript, remains challenging since there are massive numbers of miRNAs potentially regulating lineage formation^16^. In previous studies, key miRNAs were mainly revealed by the analysis of expression changes during differentiation of a single lineage (intra-lineage analysis)^9,17,18^. Following such analysis, only those key miRNAs with the greatest fold-changes were selected for further study^19–21^, while miRNAs that changed less dramatically were avoided.

Another criterion, lineage specificity, has also been used to identify key miRNAs, which alleviates the drawbacks of solely considering fold-change as the filtration criterion. Lineage-specific miRNAs have been reported previously^22,23^; several of them have been confirmed to determine cell identity^10^. For instance, *miR-124* and *miR-9*, which are neuron-specific miRNAs, have been reported to direct neuronal differentiation and regulate brain development^24^. Many lineage-specific miRNAs have been revealed by the comparison of the miRNA transcriptome (miRNAome) between terminally differentiated tissues^23,25,26^. However, few studies have examined different lineages at the earliest stages of development. Therefore, profiling of the miRNAome of the three germ layers and further lineages induced from hPSCs would allow the identification of lineage-specific miRNAs that affect the earliest cell fate decisions.

Human embryonic stem cells (hESCs) are the most frequently used hPSCs in *in vitro* studies of human development^27^, although the full-scale application of hESCs in regenerative medicine and disease modeling is hindered by ethical and technical issues^28^. Human induced PSCs (hiPSCs), which resemble hESCs, have become an alternative cell type for disease modeling and drug selection^7^. hiPSCs induced from somatic cells of individual patients enable the establishment of hiPSC banks corresponding to all HLA haplotypes. Considering the necessity of integrating information from different patients, generation of a reference dataset of miRNAome based on hiPSCs of healthy individuals that could be used for comparison with any patient-specific hiPSCs is urgently required^29^.

In this study, we performed comparisons among three lineages (inter-lineage analysis) to reveal lineage-specific miRNAs at the early stages of development. From these miRNAs, we further identified novel key miRNAs and validated their key regulatory roles in early lineage specification. To perform the inter-lineage analysis, we induced hiPSCs into three representative lineages for the three germ layers and profiled the expression changes of miRNAs during differentiation. By integrating all profiling results, we built a miRNA atlas that allows comparisons of miRNA expression across three lineages. Based on this atlas, those miRNAs with lineage-specific expressions, such as *miR-192* and *miR-372-3p*, were easily identified. After selecting key miRNA candidates by inter-lineage analysis, we conducted extensive experiments to determine whether they were true key regulators. Targets of *miR-192* and *miR-372-3p* in lineage specification were predicted computationally. Their repression on predicted targets and their regulatory effects on lineage differentiation were validated experimentally. These results strongly supported the hypothesis that new key miRNAs could be precisely identified from our atlas using inter-lineage analysis.

Taken together, we have developed the inter-lineage analysis methodology to identify key miRNAs of early hPSCs differentiation by considering lineage specificity. The inter-lineage analysis is complementary to intra-lineage analysis and allows key miRNAs with less dramatic fold-changes to be identified. Our study differs from previous studies primarily in terms of: (1) determining which miRNAs affect the very earliest stages of hPSCs differentiation (key miRNAs); (2) the development of a computational method to identify key miRNAs from lineage-specific miRNAs; and (3) the generation of an integrated miRNA expression atlas for all three germ layers and their derived lineages.

## Results

### Step-wise *in vitro* differentiation of hiPSCs into hepatocytes, nephron progenitors, and neural progenitors

**Figure 1:**
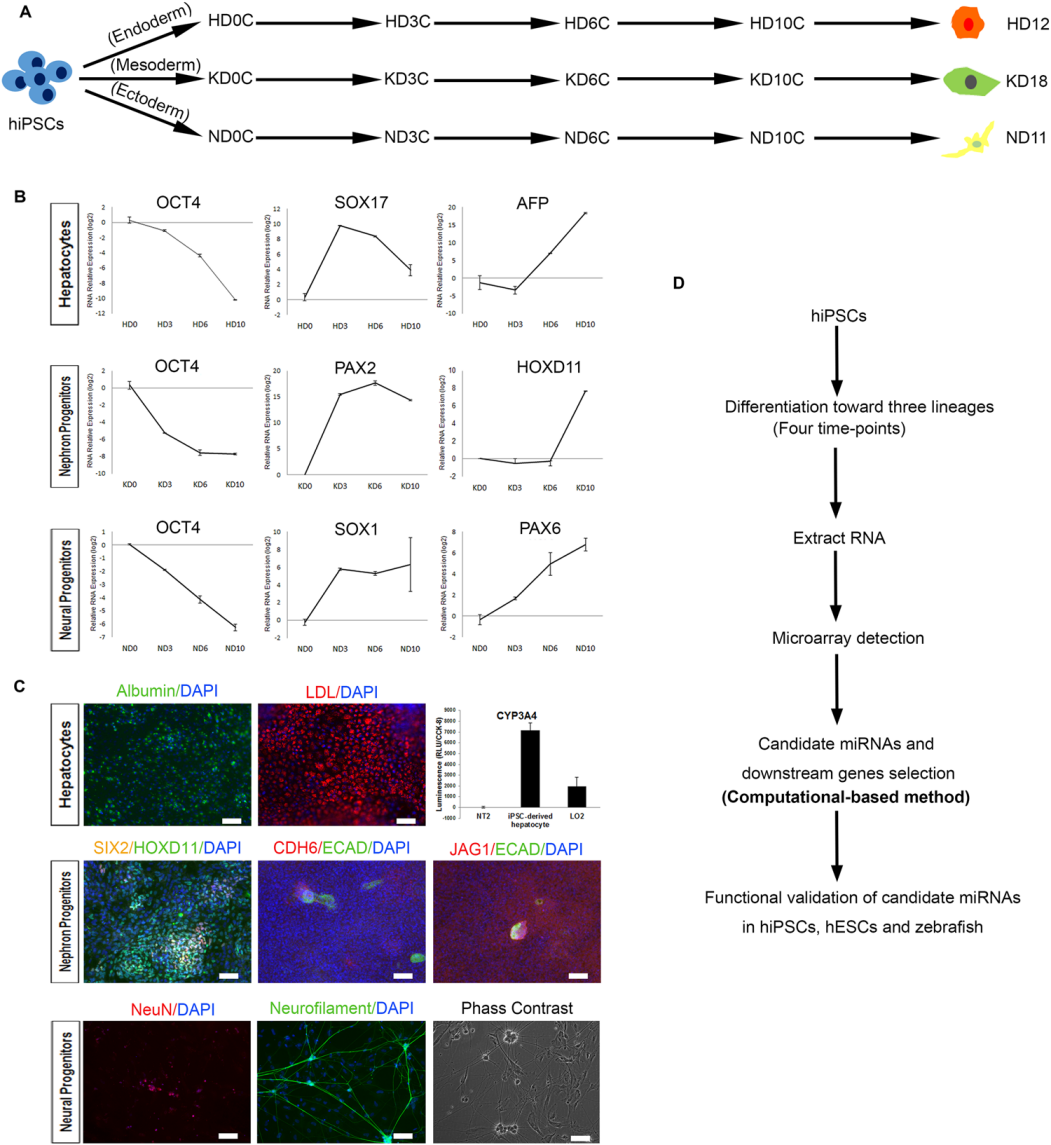
Outline of the experimental design and summary of the multi-lineage induction. (**A**) Schematic overview of hiPSC differentiation into hepatocytes, nephron progenitors, and neural progenitors. (**B**) qPCR results showing the expression tendencies of pluripotency marker (*OCT4*), markers for endoderm (*SOX17*), mesoderm (*PAX2*), ectoderm (*SOX1*), and representative markers for hepatocytes (*AFP*), metanephric mesenchyme (*HOXD11*), and neural progenitors (*PAX6*) at four time-points. Values represent means ± SD (n = 2 independent cultures for each time-point). (**C**) Functional characterization of terminal cells, including hepatocytes (12 days), nephron progenitors (18 days) and neurons (40 days). For hepatocyte differentiation, *Albumin* (green fluorescence) indicates hepatocytes; LDL uptake assay indicates the LDL receptor activity in hepatocytes; and CYP450 assays show the cytochrome P450 activity of hepatocytes. For kidney differentiation, early metanephric mesenchyme marked by SIX2 (yellow fluorescence) and HOXD11 (green fluorescence), and nephron vesicles marked by CDH6 (green fluorescence), ECAD (red fluorescence) and JAG1 (red fluorescence) were induced successfully. For neuron differentiation, the nucleus and the axons of neurons were marked by NeuN (red fluorescence) and Neurofilament (green fluorescence), respectively. Phase contrast imaging showing the morphology of an induced neuron. Scale bars represent 100 μm. (**D**) Schematic overview of the experimental design. HD: hepatocyte differentiation; KD: nephron progenitor differentiation; ND: neural progenitor differentiation.

To clarify the miRNA dynamics involved in hiPSCs differentiation, we established a multi-lineage differentiation system following published protocols that show high differentiation efficiency^30–32^. We differentiated hiPSCs into representative lineages of the three germ layers: hepatocytes for the endoderm (12 days), nephron progenitors for the mesoderm (18 days), and neural progenitors for the ectoderm (11 days), respectively (Fig. 1A and Supplementary Fig. S1A). The hiPSC line used in this study (iBC 1.2) meets all established criteria for hiPSCs, as reported in our previous studies^33,34^. The expression of representative markers for each lineage was detected by quantitative real-time PCR (qPCR) to confirm the cell identity at each time-point (Fig. 1B and Supplementary Fig. S1B–D). The expression of the pluripotent marker *OCT4* declined during differentiation of all three lineages, indicating a loss of pluripotency. Meanwhile, germ layer markers and lineage-specific markers appeared in a sequential order, as expected. Markers for hepatocytes, metanephric mesenchyme (MM), and neural progenitors were confirmed using immunofluorescent staining (IFC) analysis and flow-cytometric analysis, indicating that the purities of the three lineages were all around 50% (Supplementary Fig. S1E–K). Hepatocytes and neurons were additionally confirmed by functional assays and IFC, respectively (Fig. 1C).

### Intra-lineage analysis of differentially expressed miRNAs confirms previously reported key miRNAs

**Figure 2:**
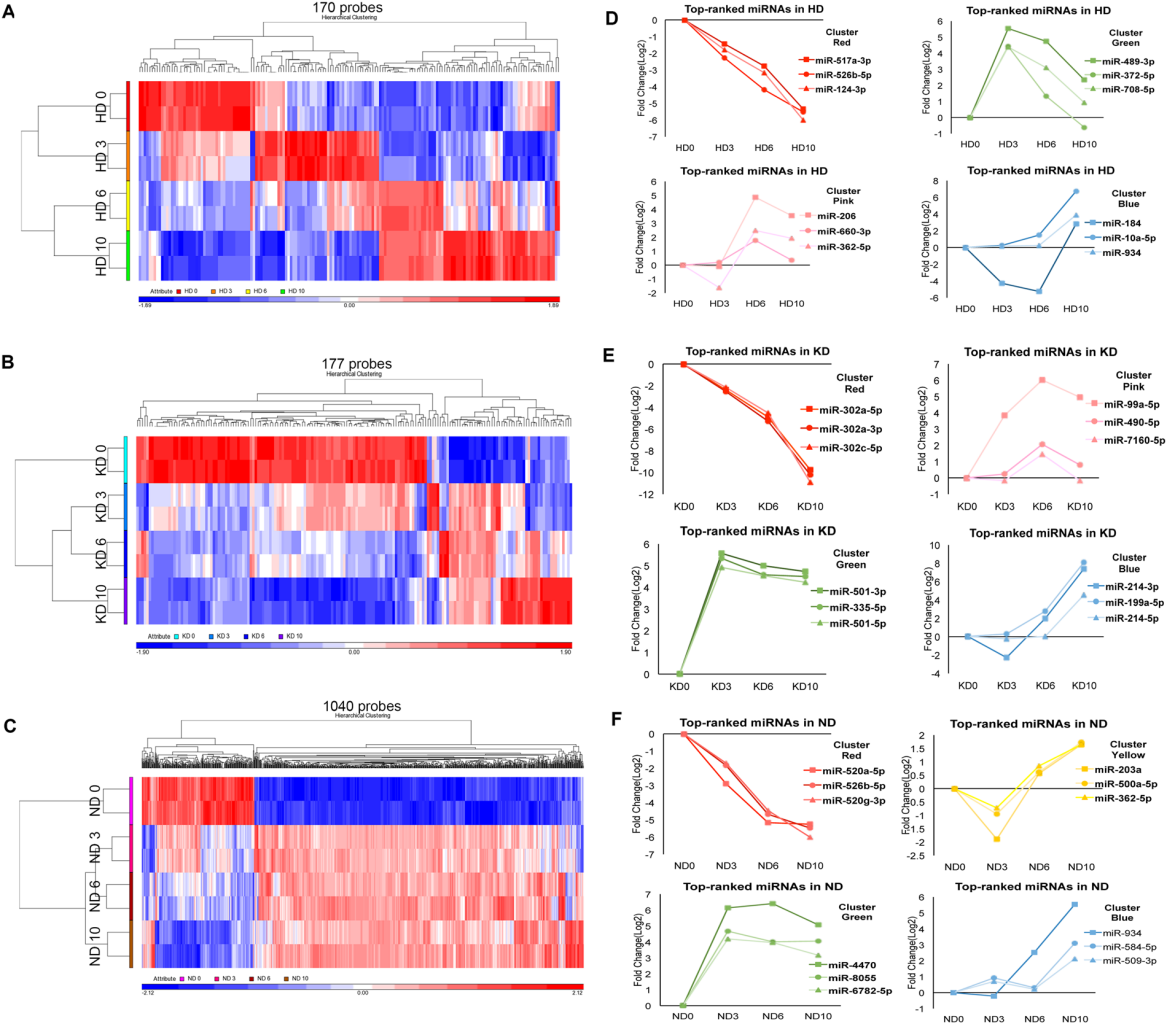
Intra-lineage analysis of differentially expressed miRNAs. (**A**–**C**) Hierarchical clustering of 170 differentially expressed small RNAs in the first ten days of HD, 177 differentially expressed small RNAs of KD, and 1,040 differentially expressed small RNAs of ND, respectively. The values of two biological replicates (indicated in color) for each time-point are shown. (**D**–**F**) The miRNAs with differential dynamics changes were plotted in different colors: small RNAs that decreased with differentiation (red); small RNAs enriched in stage 1 and 4 but downregulated at stage 2 (yellow); small RNAs increased at stage 2 but decreased at later stages (green); small RNAs enriched in stage 3 (pink); and small RNAs only increased at the latest stage (blue).

We first asked how miRNAs are differentially expressed when hiPSCs are differentiated into individual lineages (intra-lineage analysis). RNA was collected at days 0, 3, 6, and 10 (n = 2) of each lineage differentiation, as illustrated in Fig. 1D. The miRNA transcriptome (miRNAome) was examined by small RNA microarray analysis. We processed the microarray results with Partek Genomic Suite software following a standard workflow for miRNA expression. The differentially expressed small RNAs within each lineage are summarized in Supplementary Table S1. Specifically, a one-way ANOVA analysis was used to determine which small RNAs had differences in expression between time-points. Subsequent pairwise comparison between successive time-points was used to identify when small RNAs demonstrated significant differential expression (post-hoc testing, fold-change ≥2 or ≤−2, false discovery rate <0.05). We used the false discovery rate (FDR) instead of the *P* value to decrease false positives, as performed in previous studies^8,33^. These analyses identified 170, 177, and 1040 differentially expressed small RNAs in hepatocyte differentiation (HD), nephron progenitor differentiation (KD), and neural progenitor differentiation (ND), respectively (Fig. 2A–C).

Bi-directional hierarchical clustering indicated distinct expression dynamics of small RNAs when different lineages were established (Fig. 2A–C). In HD, stage 1 and 2 samples clustered together, while stage 3 and 4 samples shared a similar pattern (Fig. 2A). In contrast, in KD and ND, stage 2, 3 and 4 samples clustered together, separate from stage 1 samples (Fig. 2B,C). Within each lineage, small RNAs were separated into different groups according to their differential dynamics changes. To clearly reveal these dynamics, miRNAs with the greatest fold-changes in each group were plotted in different colors (Fig. 2D–F). To ascertain the dynamic range of the microarray platform is appropriate for reliable detection, the expression of the five most-upregulated miRNAs in each lineage were further validated by qPCR (Supplementary Fig. S2).

Using the intra-lineage analysis, we were able to recapitulate previously reported key miRNAs. Specifically, we ranked the absolute fold-changes of miRNAs during lineage differentiation. Functions of miRNAs with the largest fold-change (top 10 ranking) were identified from previously reported studies and are summarized in Table 1. As expected, most of the miRNAs with large fold-changes (9 of 10) in both HD and KD were associated with corresponding lineage establishment. For example, *miR-122*, which changes the most from HD 0 to HD 6 (positive fold-change of 1,022), has been reported to be a liver-specific miRNA that functions in the maintenance of liver phenotype and metabolism of cholesterol^35,36^.

**Table 1:** Top 10 miRNAs with previously reported development-associated functions.

miRNAs	Expression pattern	Reported functions in lineages
*miR-10a-5p*	HD 10 (up)	Liver fibrosis^62^
*miR-122-5p*	HD 6-10 (up)	Expressed in developing liver tissue^63^/Liver-specific miRNA and maintenance of adult-liver phenotype^35,36^/Cholesterol and lipid metabolism^36,64^
*miR-124-3p*	HD 3–10 (down)	Cholangiocyte proliferation^65^/Brain-specific miRNA^65,66^
*miR-146b-5p*	HD 10 (up)	Intestinal epithelial cell differentiation^67^/Visceral preadipocyte proliferation and differentiation^68^/Regulator of inflammation in epithelial cells, lung fibroblasts and others^69^
*miR-184*	HD 10 (up)	—
*miR-192-5p*/*194-3p*	HD 6–10 (up)	Liver-abundant miRNAs^70^/Intestinal epithelial cell differentiation^71^
*miR-489-3p*	HD 3 (enriched)	Increased in all germ layers, highest in the endoderm^11^
*miR-517a-3p/-526b-5p*	HD 3–10 (down)	Members of C19MC cluster, which is involved in pluripotent stem cell status^72,73^
*miR-10a-5p*	KD 6 (enriched)	Biomarkers of kidney injury^74^
*miR-10b-5p*	KD 6 (enriched)	—
*miR-181a-5p*	KD 10 (up)	Expressed in kidney tissue^75^/Hematopoietic lineage differentiation^75^
*miR-214–3p*	KD 10 (up)	Differentiation of ESCs into endothelial cells^76^/Osteogenic differentiation^77^
*miR-302a-5p*/*3p*/*-302c-5p*/*-302d-5p*	KD 10 (down)	hPSC-specific miRNAs^22^/Definitive endoderm formation^12^
*miR-371a-3p*	KD 10 (down)	hPSC-specific miRNAs^22^/Mesendoderm specification^78^
*let-7e-5p*	KD 10 (up)	Early nephrogenic differentiation^38^
*miR-10a-5p*	ND 10 (up)	Neuron differentiation^79^
*miR-181a-2-3p*	ND 3 (down)	Regulation of synaptic functions^80^
*miR-1208*	ND 6 (enriched)	—
*miR-375*	ND 6–10 (up)	Inhibition of neurite differentiation^81^/Spinal motor neuron development^82^
*miR-4470*	ND 3–6 (up)	—
*miR-520a-5p/-520g-3p/-526b-5p*	ND 3–10 (down)	Members of C19MC cluster, which is involved in pluripotent stem cell status^72,73^
*miR-664b-5p*	ND 3–10 (up)	—
*miR-934*	ND 6–10 (up)	—

In summary, the success in recapitulating many previously reported key miRNAs by intra-lineage analysis effectively supports the use of our profiling data as a reliable source for identifying key miRNAs.

### Inter-lineage analysis reveals lineage-specific miRNAs

Identification of key miRNAs by intra-lineage analysis has traditionally relied on fold-change, leading to the identification of only key miRNAs with large fold-changes. Here, we applied another criterion to identify key miRNAs, one that focuses on their differential expression across lineages in addition to considering fold-change^23,25,26^.

According to previous studies, miRNAs with expression restricted to one lineage contribute to cell identity^10^, suggesting that the expression changes of miRNAs (upregulation or downregulation) have a significant potential to specify lineages. Therefore, we hypothesized that miRNAs that specifically change at one lineage are potential candidates for key miRNAs. For convenience, we termed these miRNAs “lineage-specific miRNAs”. Here, we describe how we identify them by comparing the expression of miRNAs across lineages (inter-lineage analysis).

**Figure 3:**
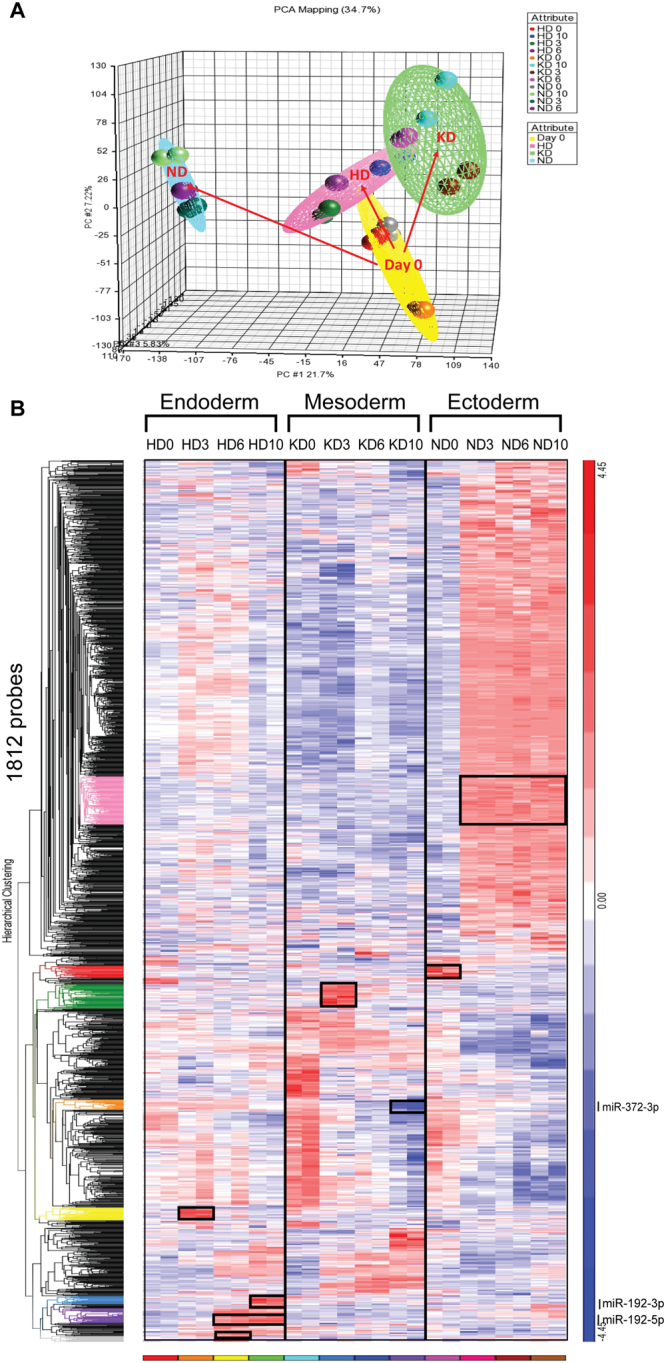
Inter-lineage analysis reveals lineage-specific miRNAs. (**A**) Principal component analysis (PCA) showing the unsupervised division of samples into a day 0 group (yellow sphere), an HD group (pink sphere), a KD group (green sphere) and a ND group (blue sphere). (**B**) Comprehensive heat map showing distinct dynamics of small RNA expression patterns during lineage specification. Colored dendrograms and black boxes indicate small RNAs that were only upregulated or downregulated in one lineage. Particularly, gray, purple, blue and yellow dendrograms indicate small RNAs that were only changed in HD. Orange and green dendrograms indicate small RNAs that were only changed in KD, Red and pink dendrograms indicate small RNAs that were only changed in ND. The newly identified key miRNAs (*miR-192-3p/5p* and *miR-372-3p*) are indicated in the dendrograms that they are included.

First, we subjected all small RNA transcriptome data (24 samples in total for all three lineages) to principal component analysis (PCA). The 24 samples automatically segregated into four groups, including undifferentiated hiPSCs with different pre-induction methods (HD 0, ND 0, KD 0, yellow sphere), differentiating hepatocytes (HD 3–10, pink sphere), differentiating nephron progenitors (KD 3–10, green sphere), and differentiating neural progenitors (ND 3–10, blue sphere) (Fig. 3A).

Next, to further show the expression patterns of small RNAs in the establishment of different lineages, we performed hierarchical clustering of all identified differentially expressed small RNAs (1,812 small RNAs) in three lineages (Fig. 3B). The bi-direction hierarchical clustering automatically resulted in several subclusters with distinct expression patterns (Fig. 3B)^37^. Within these subclusters of small RNAs, we focused primarily on miRNAs and denoted them as lineage-specific miRNAs. To clearly show these lineage-specific miRNAs in the atlas, we delineated them with black boxes in the heat-map and colored them in the dendrogram (Fig. 3B). For instance, the subcluster of miRNAs colored green in the dendrogram was specifically elevated in KD 3 but not in HD or ND (Fig. 3B). All lineage-specific miRNAs with normalized expression values are summarized in Supplementary Table S2. As expected, several of them have been previously reported to specify lineage, such as *miR-124* in ND and *let-7e* in KD^38,39^, supporting lineage specificity as an effective criterion to identify key miRNAs.

### Identification of novel key miRNAs from lineage-specific miRNAs

Lineage-specific miRNAs form a candidate set of key miRNAs (273 lineage-specific miRNAs). We next sought to select the most likely potential candidates for validation. However, since the cardinality of the candidate set (Supplementary Table S2) was large, it was difficult to evaluate every candidate and identify key miRNAs. Therefore, we focused on the HD 6–10 subcluster (purple and blue subcluster) and the KD 10 subcluster (orange subcluster). At these time-points, early lineages have formed, considering that hepatoblasts and metanephric mesenchyme are progenitors of hepatocytes and nephrons, respectively.

One straightforward method to identify key miRNAs from lineage-specific miRNAs is based on fold-change. By ranking the fold-change of lineage-specific miRNAs, we found that both *miR-192-5p* and *miR-192-3p* ranked the highest among the HD 6–10 subcluster (Fig. 3B and Supplementary Table S2), increasing our suspicion that they were candidate key miRNAs. However, selection by fold-change can only identify key miRNAs that change substantially, while it ignores those changing less dramatically. We therefore developed a computational method to expand the search range (Supplementary Fig. S3). Briefly, given that key miRNAs should regulate critical genes in specific lineages, we first searched miRNAs targeting critical genes in KD, and then intersected these identified miRNAs with lineage-specific miRNAs to obtain final key miRNA candidates (Supplementary Fig. S3A). We focused on *PKD1* and *PKD2*, which are key regulatory genes that encode Polycystin 1 (PC1) and Polycystin 2 (PC2), respectively. They are upregulated synergistically during the early stages of embryonic renal development and are important in the developing kidney for maintaining the differentiated phenotype of tubular epithelium^40^. We then asked whether there were any miRNAs contributing to kidney development via regulation of *PKD1* or *PKD2*. TargetScan generated a list of potential miRNAs for targeting *PKD1*/*PKD2*. Because *PKD1*/*PKD2* were upregulated in KD (Supplementary Fig. S3B), we searched the specifically downregulated miRNAs in KD and compared the results with the TargetScan analysis. Using this approach, we recapitulated the *miR-10*6*b~25* cluster and *miR-17~92* cluster (Supplementary Fig. S3C), which showed an increased expression tendency in a polycystic kidney disease (PKD) mouse model^41^. More importantly, the overexpression of the *miR-17~92* cluster was sufficient to produce cyst-like structures in the mouse model^41^. In addition to previously reported miRNAs, we identified *miR-372-3p*, which has well-established enrichment in hESCs but has never been reported in kidney development^42^ (Supplementary Fig. S3D). Our array results indicated that *miR-372-3p* was specifically downregulated from KD 6 to 10 (Fig. 3B). TargetScan analysis showed that both *PKD1* and *PKD2* are targeted by *miR-372-3p* (Supplementary Fig. S3E).

### Effects of novel key miRNAs on lineage specification

To experimentally validate that *miR-192* and *miR-372-3p* indeed regulate lineage specification, we performed functional characterization to determine their effects. The experimental validations were performed in both hESCs (H1) and hiPSCs (iBC 1.2) to rule out the possibility of hiPSC-specific “artifacts”.

#### Effects of miR-192 on hepatocyte differentiation

**Figure 4:**
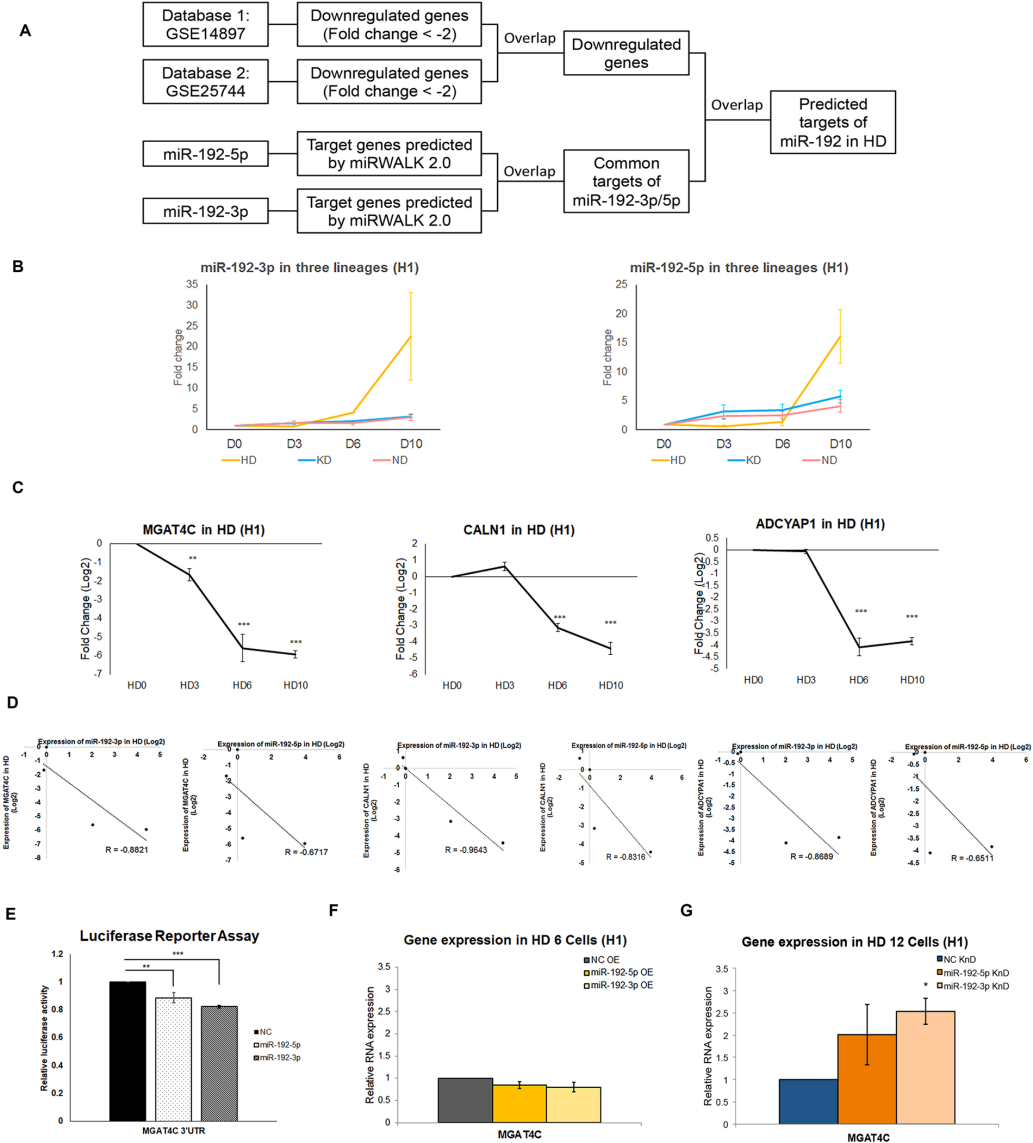
Identification of target genes of key miRNAs during hepatocyte differentiation. (**A**) Strategy for predicting common targets of *miR-192-3p* and *miR-192-5p* during HD. (**B**) TaqMan qPCR analysis confirming that *miR-192-3p*/*5p* were specifically upregulated during HD (n = 3 independent cultures for each time-point). (**C**) qPCR results showing the expression tendencies of common targets of *miR-192-3p*/*5p* during HD (n = 3 independent cultures for each time-point). (**D**) Correlation plot revealing reverse-correlations between *miR-192-3p/5p* and *MGAT4C*/*CALN1*/*ADCYAP1*, respectively. (**E**) Luciferase reporter assay confirming that *miR-192-3p*/*5p* could inhibit the 3’UTR of *MGAT4C* (n = 3 independent cultures for each group). (**F**) qPCR results showing the expression of ectodermal marker *MGAT4C* in HD 6 cells upon transfection of *miR-192-3p*/*5p* mimics (n = 3 independent cultures for each group). (**G**) qPCR results showing the expression of *MGATC4C* in HD 12 cells upon transfection of *miR-192-3p*/*5p* inhibitors (n = 3 independent cultures for each group). In (**C**), data are presented as the means ± SD. *P < *0.05*, **P < *0.01*, ***P < *0.001* for statistical comparisons between day 0 and other time-points (ANOVA plus Bonferroni’s post hoc test). In (**E**–**G**), data are presented as the means ± SD. *P < *0.05*, **P < *0.01*, ***P < *0.001* for statistical comparisons between control groups and experimental groups (ANOVA plus Bonferroni’s post hoc test). OE: overexpression; KnD: Knockdown; KD: nephron progenitor differentiation; NC: non-targeting control.

We first sought to determine what the targets of *miR-192* in HD. To predict targets of *miR-192* in a more deterministic manner, we applied bioinformatics analysis by combining published transcriptional datasets and the *in silico* target prediction (Fig. 4A). The qPCR analysis confirmed that *miR-192-5p*/*3p* were only upregulated during HD (Fig. 4B and Supplementary Fig. S4A). Due to inhibitory effects of miRNAs on target mRNAs, only downregulated genes (fold-change ≤−2) from two independent profiling studies were identified (Supplementary Fig. S4B and Table S3)^43,44^. With further filtration by miRWalk, in which the interactions between miRNAs and mRNAs could be predicted by different online tools, common targets of *miR-192-3p* and *miR-192-5p* were identified (Supplementary Fig. S4C and Table S3). By overlapping the list of downregulated genes and targets of *miR-192*, we identified 123 potential targets of *miR-192* in HD (Supplementary Fig. S4D and Table S3).

As the fold-changes of genes detected by the two datasets were modulated, we randomly selected several genes for validation in our HD samples. The qPCR analysis showed that Calneuron 1 (*CALN1*), MGAT4 family member C (*MGAT4C*) and adenylate cyclase activating polypeptide 1 (pituitary) receptor type I (*ADCYAP1R1*) were downregulated (Fig. 4C and Supplementary Fig. S4E). A negative correlation between *miR-192-5p*/*3p* and *CALN1*/*MGAT4C*/*ADCYAP1R1* was observed (Fig. 4D and Supplementary Fig. S4F). Since *CALN1* is a human brain-specific gene, whereas *MGAT4C* is normally expressed in mesodermal tissues like the kidney and *ADCYAP1R1* encodes the receptor for peptide signals in response to stress in the brain^45–47^, we reasoned that *miR-192* may promote HD via repressing genes normally expressed in the other two germ layer-derived lineages.

Given that both microarray and qPCR analysis detected a significant downregulation of *MGAT4C*, we further examined the molecular effects of *miR-192* on this gene. Luciferase reporter assays showed that *miR-192-5p* and *miR-192-3p* mimics could suppress the *MGAT4C* 3′UTR (Fig. 4E). In addition to this, we also examined the expression change of *MGAT4C* upon disturbance of *miR-192* expression. When H1 cells were transfected with *miR-192-5p* and *miR-192-3p* mimics from HD 0–6, *MGAT4C* transcript levels were moderately decreased (Fig. 4F). However, when *miR-192-5p* and *miR-192-3p* inhibitors were transfected from HD 6–12, we observed an upregulation of the *MGAT4C* transcript levels at HD 12 (Fig. 4G). Similar results were obtained in iBC 1.2 cells (Supplementary Fig. S4G,H).

**Figure 5:**
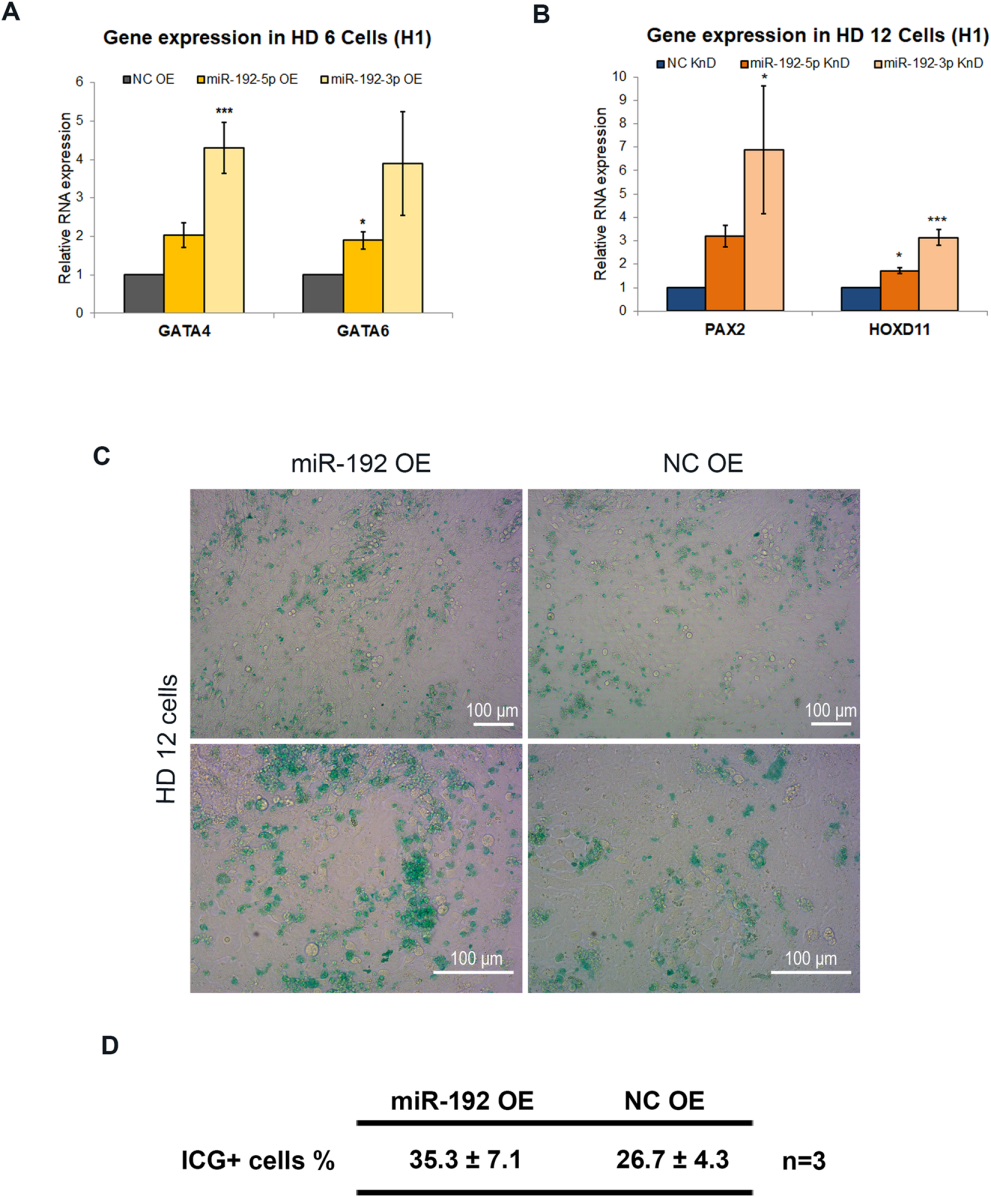
Regulation of lineage-specific gene expression by lineage-specific miRNAs during hepatocyte differentiation. (**A**) qPCR results showing the expression of definitive endoderm markers *GATA4* and *GATA6* in HD 6 cells with transfection of *miR-192-3p*/*5p* mimics (n = 3 independent cultures for each group). (**B**) qPCR results showing the expression of intermediate mesoderm marker *PAX2* and metanephric mesenchyme marker *HOXD11* in HD 12 cells upon transfection of *miR-192-3p*/*5p* inhibitors (n = 3 independent cultures for each group). (**C**) HD 12 cells were examined by indocyanine green staining (ICG). The cells transfected with *miR-192* mimics (left column) and scramble controls (right column) were compared (Scale bars represent 100 μm). (**D**) Table showing the percentage of ICG^+^ cells at HD 12 with the transfection of *miR-192* mimics and non-targeting controls (data are presented as the means ± SD. n = 3 independent cultures for each group). In (**A**,**B**), data are presented as the means ± SD. *P < *0.05*, **P < *0.01*, ***P < *0.001* for statistical comparisons between control groups and experimental groups (ANOVA plus Bonferroni’s post hoc test). OE: overexpression; KnD: Knockdown; NC: non-targeting control.

Next, we determined the regulatory effects of *miR-192* on lineage specification. We examined the expression changes of lineage-specific genes while modulating *miR-192* expression during HD. When H1 cells were transfected with *miR-192-5p*/*3p* mimics from HD 0–6, *GATA4* and *GATA6*—which are expressed during definitive endoderm differentiation^48^—were elevated with the overexpression *of miR-192-5p*/*3p* (Fig. 5A). Conversely, the intermediate mesoderm marker *PAX2* and the MM marker *HOXD11* were significantly increased upon inhibition of *miR-192-5p*/*3p* from HD 6–12 (Fig. 5B and Supplementary Fig. S5), supporting that *miR-192-5p*/*3p* repress mesodermal differentiation.

To demonstrate the effects of *miR-192* on HD, we also determined the functional characteristics of HD 12 cells when modulating the expression of *miR-192*. Indocyanine green (ICG) is a non-toxic organic anion that can be eliminated exclusively by mature hepatocytes^49^. Therefore, uptake of ICG is used to measure the hepatic functions. When H1 cells were transfected with *miR-192-5p mimics*, ICG analysis showed that the percentage of ICG-positive cells at HD12 was increased substantially (Fig. 5C,D).

Taken together, we concluded that *miR-192* is likely a key miRNA that promotes endodermal hepatic differentiation while inhibiting the formation of mesoderm.

#### Effects of miR-372-3p on the expression of KD-specific genes

**Figure 6:**
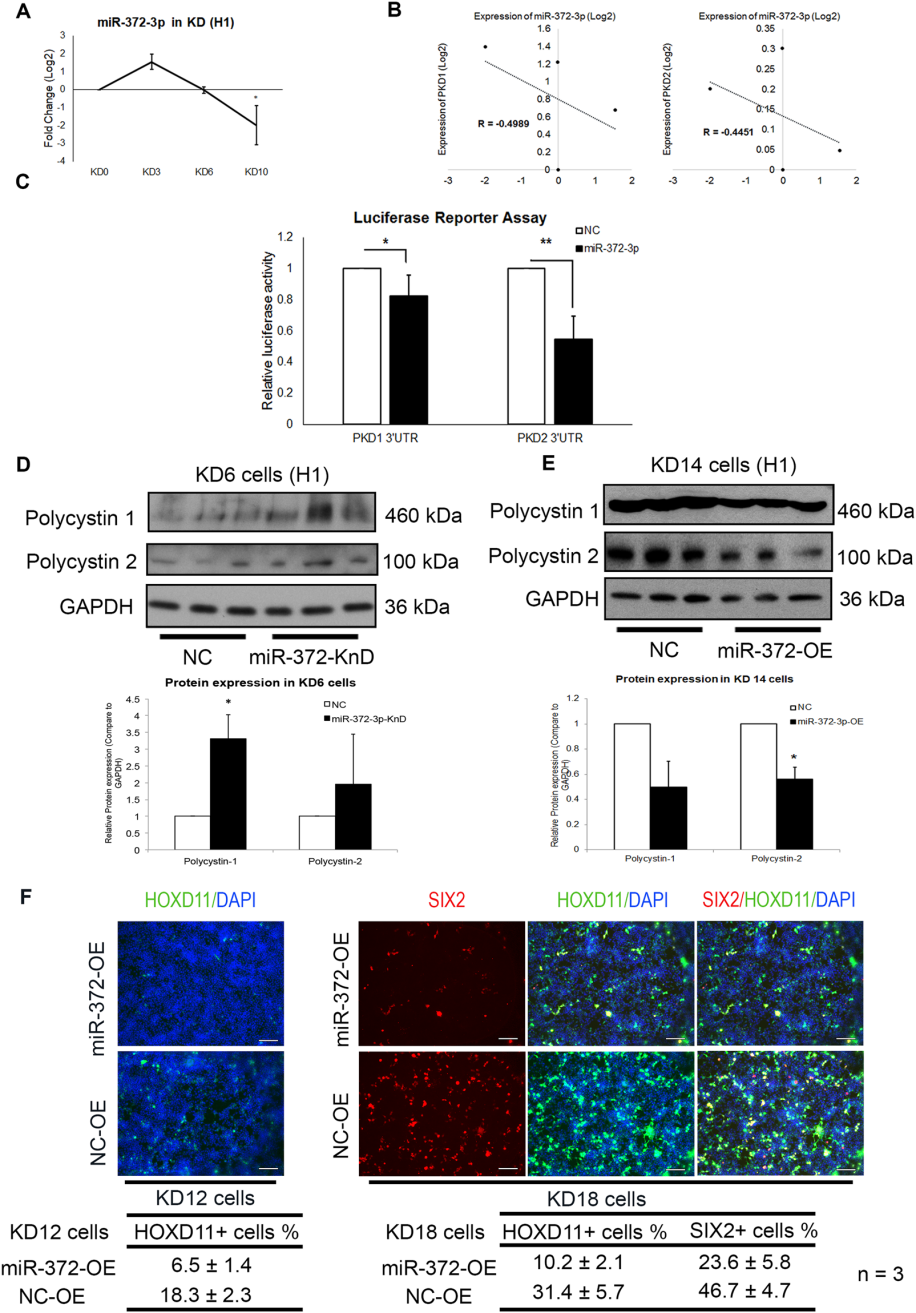
Regulation of *PKD1*/*PKD2* expression by lineage-specific miRNAs during nephron progenitor differentiation. (**A**) Taqman qPCR analysis showing the expression tendency of *miR-372-3p* during KD (n = 3 independent cultures for each time-point). (**B**) Correlation plot revealing reverse-correlations between *miR-372-3p* and *PKD1*/*PKD2*, respectively. (**C**) Luciferase reporter assay results confirming that *miR-372-3p* could inhibit the 3′UTR of both *PKD1* and *PKD2* (n = 4 independent cultures for each group). (**D**,**E**) Western blot results (upper panel) showing expression of Polycystin 1 and Polycystin 2 in KD 6 cells (**D**) with transfection of *miR-372-3p* mimics or non-targeting controls, and expression of Polycystin 1 and Polycystin 2 in KD 14 cells (**E**) upon transfection of *miR-372-3p* inhibitors or non-targeting controls. Normalized protein expressions of Polycystin 1 and Polycystin 2 are shown in lower panel (**D**,**E**). The expression of NC group of each experiment is set as 1. n = 3 independent cultures for each group. (**F**) KD 12 cells (left column) and KD 18 cells (right column) were examined by immunofluorescent staining (IFC) analysis. The cells transfected with *miR-372-3p* mimics (upper row) and scramble controls (lower row) were compared (Scale bars represent 200 μm). Table showing the percentage of HOXD11^+^ cells at KD 12, SIX2^+^ cells and HOXD11^+^ cells at KD 18, with the transfection of *miR-372-3p* mimics and non-targeting controls (data are presented as the means ± SD. n = 3 independent cultures for each group). In (**A**), data are presented as the means ± SD. *P < *0.05*, **P < *0.01*, ***P < *0.001* for statistical comparisons between day 0 and other time-points (Paired two-tailed *t*-test). In (**C**–**E**), data are presented as the means ± SD. *P < *0.05*, **P < *0.01*, ***P < *0.001* for statistical comparisons between control groups and experimental groups (Paired two-tailed *t*-test). OE: overexpression; KnD: Knockdown; KD: nephron progenitor differentiation, NC: non-targeting control.

To assess the regulatory roles of *miR-372-3p* in KD, we first performed qPCR analysis to confirm the downregulation of *miR-372-3p* (Fig. 6A and Supplementary Fig. S6A). A negative correlation between *miR-372-3p* and *PKD1/PKD2* was identified (Fig. 6B and Supplementary Fig. S6B). Next, we performed luciferase reporter assays and found that the 3′UTRs of *PKD1*/*PKD2* were significantly inhibited by *miR-372-3p* (Fig. 6C). Since miRNAs can lead to mRNA decay or inhibit translation, we further measured the protein levels of PC1/PC2. Knockdown of *miR-372-3p* during early nephron differentiation (KD 0–6) resulted in increased PC1 and PC2 protein levels (Fig. 6D and Supplementary Fig. S6C). Conversely, overexpression of *miR-372-3p* at KD 8–14 resulted in decreased PC1 and PC2 protein levels (Fig. 6E and Supplementary Fig. S6D).

To demonstrate the effects of *miR-372-3p* on KD, we also determined the efficiency of KD when modulating the expression of *miR-372-3p*. Briefly, we measured the expression of cellular markers that are important for kidney development including SIX2 and HOXD11. IFC analysis showed that the percentage of cells expressed the early metanephric marker HOXD11 at KD 12 was decreased substantially when H1 cells were transfected with *miR-372-3p* mimics (Fig. 6F). Similarly, the percentage of cells that eventually expressed both HOXD11 and the nephric marker SIX2 at KD 18 was decreased upon the transfection (Fig. 6F), indicating that *miR-372-3p* inhibits the formation of nephron progenitors.

Taken together, these experiments validated that *miR-372-3p* is a key miRNA. According to intra-lineage analysis (Supplementary Table S1), *miR-372-3p* expression was not changed significantly; the change from KD 0 to KD 3 was ranked 99^th^ among 177 small RNAs. As a result, it was difficult to identify *miR-372-3p* as a key miRNA by intra-lineage analysis. In contrast, inter-lineage analysis combined with bioinformatics analysis allowed us to identify *miR-372-3p* as a key miRNA, supporting the use of inter-lineage analysis to discover novel key miRNAs.

## Discussion

MiRNAs have been found to act as critical regulators in lineage specification. Identification of key miRNAs regulating hPSCs differentiation has become a leading research topic. Intra-lineage analysis, which solely considers miRNAs with large fold-changes, has frequently been used to identify key miRNAs. More recently, inter-lineage analysis has been used to identify key miRNAs with lineage specificity. However, investigation of human-specific key miRNAs at the earliest stages of development has still been hindered by a lack of human samples; hPSC-derived lineages have been developed to overcome this challenge. Herein, we applied inter-lineage analysis to hPSC-derived lineages to identify key miRNAs with lineage specificity.

To identify key miRNAs by inter-lineage analysis, we built a miRNA atlas for human *in vitro* early cell lineage specification. This atlas depicts the dynamic expression of miRNAs during the three germ layer formation and lineage differentiation. With this atlas, we can easily identify lineage-specific miRNA in a readily-visualized manner. In this study, we identified *miR-192* and *miR-372-3p* as key miRNAs regulating HD and KD, respectively. To further validate *miR-192* and *miR-372-3p* as key miRNAs, we performed comprehensive experiments to validate their regulatory functions in HD and KD. First, we confirmed that *miR-192* and *miR-372-3p* directly repressed their downstream targets. Next, we modulated their expression during lineage differentiation. The disruptions skewed lineage formation by affecting the expression of lineage markers. Particularly, our data showed that the overexpression of *miR-192* increased the expressions of endodermal markers *GATA4*/*6*, while knockdown of *miR-192* increased the expression of mesodermal marker *PAX2*. Given that endoderm and mesoderm share the same progenitors, perhaps the upregulation of *miR-192* potentially affects the segregation of endodermal and mesodermal cell fate, leading to an increase in the formation of hepatic endoderm at the expense of mesodermal formation^50^. Finally, we performed functional characterization of *miR-192* and *miR-372-3p* in lineage differentiation, establishing that *miR-192* and *miR-372-3p* affect HD and KD, respectively. These validations prove that *miR-192* and *miR-372-3p* are truly key miRNAs.

In addition to revealing lineage-specific miRNAs, our miRNA atlas can also be used as a roadmap for lineage distinction, as it reveals lineage-specific patterns of miRNA expression. In HD, for example (Fig. 2A), four clusters represented small RNAs highly expressed at HD 0, 3, 6 and 10, respectively. Another cluster remained high from HD 6 to 10. Similar to HD, KD was also characterized by stage-specific clusters (Fig. 2B). In ND, however, the pattern was remarkably different (Fig. 2C). We observed two major clusters, the expression levels of which were dramatically changed as early as ND 3. Meanwhile, this atlas clearly identified many small RNAs that changed specifically. For instance, the miRNAs that increased from ND 3 to ND 10 (purple dendrogram) had low expression in the other two lineages. These lineage-specific clusters can be used as markers to identify lineages. Notably, since the hiPSCs were induced to generate HD 0 cells, KD 0 cells and ND 0 cells with different pre-induction methods (Supplementary data), the expression patterns of miRNAs at day 0 were variable in different lineages (Fig. 3).

Table 1 shows that the top 10 miRNAs in ND were much less likely to have known functions in neural development compared to those in HD and KD. This might be due to the increasing complexity of development from small mammals to primates, especially in the neuronal system^51^. More miRNAs are involved in ND of primates during evolution, whereas the majority of developmental studies have been performed in small mammals^52^, leading to many neuron-specific primate miRNAs that have remained functionally undescribed, requiring specific studies with primate samples^53^.

In addition to human developmental processes, our miRNA atlas can be applied to the study of complex congenital disorders. Mutations in the *PKD1*/*PKD2* genes or abnormal expression of PC1/PC2 are known to cause the formation of renal cysts^54–56^. We newly identified the regulation of *miR-372-3p* on *PKD1*/*PKD2*, indicating the possible association of *miR-372-3p* with polycystic kidney disease for the first time. Moreover, the key miRNAs identified from our atlas are also potential markers and therapeutic targets for various diseases, as treatments in which the miRNA activity is inhibited are in various stages of preclinical or clinical trials^57,58^. There was a differential change of *miR-372-3p* expression between hiPSC- and hESC-derived KD 3 cells that might be due to known differences in microRNA expression between these two hPSC types^59^. However, after KD 3, *miR-372-3p* expression decreased in both hPSC-derived cells. Together with its inhibitory effects on PC1/PC2 at KD 6, the substantial decline in *miR-372-3p* expression levels after KD 3 was sufficient to increase the expression levels of PC1/PC2, despite the differences at earlier time-points.

Importantly, we selected hiPSCs but not hESCs for the construction of the atlas. Considering the necessity of integrating information from different patients, we attempted to produce a reference dataset based on hiPSCs of healthy individuals that could be used for comparison with any patient-specific hiPSCs^29^. Given that hESCs are a widely used model for experimental subjects^27^, we used these for testing to provide fair comparisons between hiPSCs and hESCs and to confirm the reliability of our dataset. Compatible expression patterns of the most upregulated miRNAs in hiPSC- and hESC-derived cells (Supplementary Fig. S2) suggested that differential open chromatin states did not affect the expression patterns of important miRNAs. The hiPSCs were reprogrammed from male fibroblasts, which is the most common model despite the existence of male skin lineage memories. Given that the expression profile of miRNAs is different between women and men in many somatic tissues^60^, our miRNA atlas is more applicable for studies of male tissues.

To construct the atlas, we followed three well-established protocols to establish the multi-lineage differentiation systems. After successful inductions of HD, KD, and ND, we measured the differentiation efficiencies using IFC and flow-cytometric analysis to, by which the purities of desired somatic cell types were indicated up to 70%. These results suggested that the data outputted from these derived cells represents a mixture of miRNAs changed from wanted cells and unwanted cells. Therefore, to construct a more reliable atlas, an analysis of pure population will be important and necessary in the future.

A statistical test with non-adjusted *P* values has an increased chance of drawing false conclusions when multiple tests are performed^61^. As a result, a non-adjusted *P* < 0.05 is more likely to appear in our case when testing many outcomes from a single intervention. Herein, we adopted a more stringent criterion for multiple testing, the FDR-adjusted *P* value^61^. Compared to the non-adjusted *P* value, fewer but more reliable results have been generated by FDR. Using HD as an example, 826 and 170 differentially expressed miRNAs could be identified with a *P* < 0.05 and an FDR < 0.05, respectively. As a tradeoff, an FDR < 0.05 excluded several important candidates, such as *miR-375*; FDR was too conservative in this case. When we ran the analysis with a *P* < 0.05, *miR-375* was the number 1 upregulated miRNA (fold-change = 104, *P* = 0.001291) from HD 0–3, which is consistent with its implicated functions in the endodermal formation^8,11–15^. Therefore, the statistical criterion using the *P* value can be applied to our dataset when more candidate miRNAs need to be identified.

In summary, our inter-lineage analysis generated a list of lineage-specific miRNAs during hiPSC differentiation. This list can serve as a source for the identification of key miRNAs and further investigation of the molecular mechanisms underlying hPSC differentiation. Moreover, the integrated miRNA expression atlas can further serve as a reference for studying human developmental processes and human congenital disease.

## Materials and Methods

### hiPSC culture and differentiation

iBC 1.2 is a hiPSC line that has been previously reported. iBC 1.2 was generated from a “normal” individual by a viral infection with genetic integration. H1 is a widely used hESC line. Both iBC 1.2 and H1 are male PSC lines. hiPSCs and hESCs were maintained with mTeSR medium (05870, StemCell Technologies, Vancouver, BC, Canada) on Matrigel matrix (354277, Corning, NY, USA)-coated plates and passaged with dispase (354235, Corning, NY, USA) every 4–6 days. They were induced according to previously established protocols for hepatocytes, nephron progenitors, and neural progenitors. The details of the three lineage induction procedures are described in the Supplemental Methods.

### Immunofluorescence Microscopy

Cells were fixed in 4% formaldehyde for 20 minutes and permeabilized with 0.1% Triton X-100 for 45 min, after which the cells were blocked with 1% BSA and 4% goat serum in PBS for 45 min. The cells were then incubated with primary antibodies at 4 °C overnight and with secondary antibodies at room temperature for 1 hour. Finally, nuclei were stained with DAPI. The details regarding the antibodies and dilution ratio are listed in the Supplemental Information. Cells were imaged with a Nikon Ti-E Live-Cell Imaging System.

### RNA extraction and real-time quantitative PCR

Total RNA, including miRNAs, were extracted from cells using the RecoverAll™ Total Nucleic Acid Isolation Kit for FFPE (AM1975, Thermo Fisher Scientific). RNA quantitation was performed using a NanoDrop 2000 Spectrophotometer (Thermo Scientific). RNA quality was examined using an Agilent 2100 Bioanalyzer. For cell samples prepared for microRNA microarray detection, an RNA integrity number larger than 9.0 was accepted. For gene expression analysis, cDNA was synthesized with PrimeScirpt™ Reverse Transcriptase (RR036A, Takara). SYBR Green master mix was used for PCR in a QuantStudio™ 7 Flex System (Life Technologies). *GAPDH* and 18 S RNA were used as internal controls. For microRNA expression analysis, RNA was reverse-transcribed with a TaqMan MicroRNA Reverse Transcription Kit, and amplification was performed using TaqMan probes with Taqman Universal Master Mix II, without UNG (Thermo Fisher Scientific). *RNU6B* was used as an endogenous control for normalization of miRNAs. The samples were plotted relative to control samples (HD0, KD0, and ND0), and the standard deviation of at least three measurements was calculated.

### MicroRNA microarray analysis and data processing

MiRNA microarray expression data were acquired on an Affymetrix miRNA 4.0 platform (2,578 mature miRNAs, 2,025 stem-loop miRNAs, and 1,996 other small RNAs), using 500 ng total RNA per sample. Microarray data were deposited in the Gene Expression Omnibus (http://www.ncbi.nlm.nih.gov/geo/) under accession number GEO: GSE97952. miRNA expression data were then analyzed in the Partek® Genomics Suite® following the workflow for miRNA microarray analysis to detect differentially expressed miRNAs between samples. MicroRNAs of interest were filtered using a cut-off fold-change value of ≥2 or ≤−2 with an FDR <0.05. Both hierarchical clustering results and a heat map were generated with the Partek® Genomics Suite® platform. miRNA target prediction algorithms were performed with TargetScanHuman 6.2 (http://www.targetscan.org/vert_61/) and miRWALK (http://zmf.umm.uni-heidelberg.de/apps/zmf/mirwalk2/). The accession projects supporting the prediction of downstream targets of *miR-192* are GSE14897 and GSE25744.

### Luciferase reporter assay

50,000 HEK 293 cells were plated in wells of 24-well plates 24 h before transfection. Reporter plasmids with *MGAT4C*, *PKD1*, *PKD2* 3′UTR were purchased from GeneCopoeia. 10 pmol synthetic miRNA mimics of specific miRNAs, including *miR-192-3p*, *miR-192-5p*, and *miR-372-3p* or non-targeting control (GenePharma, Shanghai, China), and 200 ng reporter plasmids were co-transfected with 1 μl Lipofectamine 2000 according to the manufacturer’s instructions. The medium was changed to DMEM supplemented with 10% FBS for 18 h post-transfection. At 48–72 h post-transfection, media were collected for analysis. Luciferase activities were determined with a Secrete-Pair™ Dual Luminescence Assay Kit (SPDA-D010, GeneCopoeia, Rockville, MD, USA).

### Knockdown and overexpression of miRNAs

Mimics of non-targeting control, *miR-192-3p*, *miR-192-5p*, and *miR-372-3p* miRNAs were purchased from GenePharma. Inhibitors of negative control, *miR-192-3p*, *miR-192-5p*, and *miR-372-3p* were purchased from Thermo Fisher Scientific. Cells cultured in 12-well plates were transfected with either mimics or inhibitors. First, 12.5 pmol mimics or inhibitors were diluted in 80 μl Opti-MEM. Second, 3.5 μl Lipofectamine RNAiMAX reagent was diluted in 80 μl Opti-MEM. Third, the Opti-MEM containing miRNA mimics and the Opti-MEM containing Lipofectamine® RNAiMAX Reagent were mixed at a ratio of 1:1. The mixture was incubated at room temperature for 20 minutes and added to the differentiated cells. The medium was changed the next day. Transfection was performed every two days three times. Then, the transfected cells were collected for further analysis.

### Western blot analysis

Cells were lysed in RIPA buffer with 2% proteinase inhibitor. 30 μg of total proteins were resolved on 4–20% precast polyacrylamide gels (4561096, Bio-Rad, Hercules, CA, USA), and transferred to PVDF membranes (ISEQ. 00010, Merck Millipore, Darmstadt, Germany). After transfer, the membranes were blocked with 5% Blotting-grade Blocker (1706404, Bio-Rad, Hercules, CA, USA) in PBS-T for 1 hour, and incubated with specific primary antibodies in the blocking solutions overnight at 4 °C. The membranes were then washed with PBST three times and incubated with secondary antibodies for 1 hour at room temperature. Then, the antibodies were detected using an ECL HRP substrate system (K-12045-D20, Advansta, Menlo Park, CA, USA). The protein band intensities were quantified using Image Lab software. Information regarding the antibodies is shown in the Supplemental Information.

### Statistical analysis

For microarray analysis, one-way ANOVA testing was used to determine which small RNAs had significant differences in expression between time-points. Subsequent pairwise comparisons between successive time-points were used to identify when small RNAs demonstrated significant differential expression (post-hoc testing, fold-change ≥2 or ≤−2, false discovery rate <0.05). For qPCR analysis, luciferase reporter assays and Western blotting analysis, statistical significance was determined using one-way ANOVA followed by Bonferroni’s post-hoc testing. A paired two-tailed Student’s *t*-test was performed when only two groups were compared. A single asterisk indicates *P* < 0.05, a double asterisk indicates *P* < 0.01, and a triple asterisk indicates *P* < 0.001. In all Figures, the mean ± SD is plotted.

### Data availability statement

All data generated or analysed during this study are included in this published article (and its Supplementary Information files).

## Electronic supplementary material

Supplementary Information

Table S1

Table S2

Table S3
