# Supplementary material for: Comparison of multi-lineage differentiation of hiPSCs reveals novel miRNAs that regulate lineage specification
Source: Sci Rep. 2018 Jun 25;8:9630. doi: 10.1038/s41598-018-27719-0 (PMC6018499; doi:10.1038/s41598-018-27719-0)
Supplement: Supplementary file 1 — Supplementary Information [file 41598_2018_27719_MOESM1_ESM.pdf]

Title Page

Comparison of multi-lineage differentiation of hiPSCs reveals novel miRNAs that regulate lineage specification

Running Title: Identification of novel key miRNAs by intra-lineage comparison

Lu Li[1,#a], Kai-Kei Miu[1], Shen Gu[1, #b], Hoi-Hung Cheung[1,*], Wai-Yee Chan[1,*]

[1] CUHK-CAS GIBH Joint Research Laboratory on Stem Cell and Regenerative Medicine, School of Biomedical Sciences, the Chinese University of Hong Kong, Shatin, N.T., Hong Kong SAR;

[#a] Current Address: School of Pharmacy, University of Southern California, Los Angeles, CA, USA;

[#b] Current Address: M&H Genetics / Baylor Genetics Laboratories, Baylor College of Medicine, Houston, TX, US

[*] Corresponding author, E-Mail: chanwy@cuhk.edu.hk (W.Y.C.); cheunghh@cuhk.edu.hk (H.H.C.); Tel.: +852-3943-1383; Fax: +852-2603-7902.

Key words: microRNA; microarray; lineage differentiation; functional annotation; human pluripotent stem cells; miR-192; miR-372-3p

Supplemental Figure Legends

S1 Figure. Verification of establishment of three-lineage differentiation system.

(A) Schematic illustration of the procedure to generate hepatocytes, nephron progenitors, and neural progenitors.

(B-D) qPCR results showing the mRNA expression level change of pluripotency marker (*SOX2*), markers for endoderm (*FOXA2*), mesoderm (*MIXL1*, *T*, *LHX1*), ectoderm (*FOXG1*), and representative markers for hepatocytes (*Albumin*) and metanephric mesenchyme (*SIX2*). Values are means ± SD (n = 2 independent cultures for each time-point).

(E, G and I) Immunofluorescence staining (IFC) showing the expression of markers for terminal cells, including hepatocytes marker (Albumin) for HD 12 cells, metanephric mesenchyme (HOXD11) for KD 18 cells, neural progenitor marker (PAX6) for ND 11 cells (Scale bars, 100 μm).

(F, H and J) Representative flow-cytometric diagrams of HD 12 cells transfected with an Albumin reporter, KD 12 cells stained with SIX2 antibody, and ND 11 cells stained with FOXG1 antibody.

(K) Tabular presentation of the percentage of $Albumin^{+}$ cells at HD 12, $HOXD11^{+}$ cells and $SIX2^{+}$ cells at KD 18, $PAX6^{+}$ cells and $FOXG1^{+}$ cells at ND 11 (data are presented as mean ± SD. For IFC analysis, n=5 fields in total from 3 independent cultures).

HD: hepatocyte differentiation; KD: nephron progenitor differentiation; ND: neural progenitor differentiation.

S2 Figure. Validation of the expression of Top 5 upregulated miRNAs in HD, KD and ND.

(A)(C)(E) qPCR results showing the expression tendencies of the 5 most upregulated miRNAs (indicated by array) in iBC 1.2-derived HD, KD, and ND, respectively (n = 3 independent

cultures for each time-point). Full lines represent the expression tendencies of miRNAs measured by qPCR. Dot lines represent the expression tendencies of miRNA measured by microarray. Data are presented as mean ± SD.

(B)(D)(F) qPCR results showing the expression tendencies of the 3 most upregulated miRNAs (indicated by array) in H1-derived HD, KD, and ND, respectively (n = 3 independent cultures for each time-point). Full lines represent the expression tendencies of miRNAs measured by qPCR. Dot lines represent the expression tendencies of miRNA measured by microarray. Data are presented as mean ± SD.

S3 Figure. Identification of novel key miRNAs from lineage-specific miRNAs.

(A) Schematic illustration of the process of identifying regulator miRNAs of *PKD1/PKD2* during KD.

(B) qPCR results showing the expression tendencies of *PKD1/PKD2* during KD (n = 3 independent cultures for each time-point).

(C) Microarray results showing the expression tendencies of three members of the *miR-17~92* cluster, and two members of the *miR-106b~25* cluster.

(D) Microarray results showing the expression tendencies of *miR-372-3p*.

(E) Prediction of TargetScan 6.2 software showing that *miR-372-3p* can target the 3'UTR of *PKD1* and *PKD2*.

In (B), data are presented as the means ± SD. $*P < 0.05$, $**P < 0.01$, $***P < 0.001$ for statistical comparisons between day 0 and other time-points (ANOVA plus Bonferroni's post hoc test).

S4 Figure. Effects of *miR-192* on targets during hepatocyte differentiation derived from iBC1.2.

(A) TaqMan qPCR confirming that *miR-192-3p/5p* were specifically upregulated during HD

derived from hiPSCs (iBC 1.2) (n = 3 independent cultures for each time-point).

(B-D) Bioinformatics analysis to predict the common targets of *miR-192-3p* and *miR-192-5p* in HD. 135 downregulated genes in HD (B) were identified by the intersection of downregulated genes reported from two independent studies. 16523 common targets of *miR-192-3p* and *miR-192-5p* (C) were calculated by miRWalk algorithm. 123 common targets of *miR-192-3p* and *miR-192-5p* in HD (D) were identified by the intersection of gene lists generated from (B) and (C).

(E) qPCR results showing the expression tendency of *MGAT4C* during HD derived from iBC 1.2 (n = 3 independent cultures for each time-point).

(F) The correlation plot revealing reverse-correlation between *MGAT4C* and *miR-192-3p/5p* during HD derived from iBC1.2, respectively.

(G) qPCR results showing the expression of *MGAT4C*, in HD 6 cells upon transfection of *miR-192-3p/5p* mimics (n = 3 independent cultures for each group).

(H) qPCR results showing the expression of *MGATC4C* in HD 12 cells upon transfection of *miR-192-3p/5p* inhibitors (n = 3 independent cultures for each group).

In (A and E), data are presented as mean ± SD. $*P < 0.05$, $**P < 0.01$, $***P < 0.001$ for statistical comparisons between day 0 and other time-points (ANOVA plus Bonferroni's post hoc test). In (G and H), data are presented as mean ± SD. $*P < 0.05$, $**P < 0.01$, $***P < 0.001$ for statistical comparisons between control groups and experimental groups (ANOVA plus Bonferroni's post hoc test).

OE: overexpression; KnD: Knockdown; KD: nephron progenitor differentiation, NC: non-targeting control.

S5 Figure. Effects of novel key miRNAs on hepatocyte differentiation derived from iBC1.2.

(A) qPCR results showing the expression of intermediate mesoderm (IM) marker *PAX2* and metanephric mesenchyme (MM) marker *HOXD11* in HD 12 cells derived from iBC1.2 upon transfection of *miR-192-3p/5p* inhibitors (n = 3 independent cultures for each group). Data are presented as mean ± SD. $^{*}P < 0.05$, $^{**}P < 0.01$, $^{***}P < 0.001$ for statistical comparisons between control groups and experimental groups (ANOVA plus Bonferroni's post hoc test).

KnD: Knockdown.

S6 Figure. Effects of novel key miRNAs on nephron progenitor differentiation derived from iBC1.2.

(A) qPCR showing the tendency *miR-372-3p* during KD derived from iBC 1.2 (n = 3 independent cultures for each time-point).

(B) The correlation plot revealing reverse-correlation between *miR-372-3p* and *PKD1/PKD2* during KD derived from iBC 1.2.

(C and D) Western blot results (upper panel) showing expression of Polycystin 1 and Polycystin 2 in KD 6 cells derived from iBC 1.2 (C) with transfection of *miR-372-3p* mimics or non-targeting controls, and expression of Polycystin 1 and Polycystin 2 in KD 14 cells derived from iBC 1.2 (D) upon transfection of *miR-372-3p* inhibitors or non-targeting controls. Normalized protein expressions of Polycystin 1 and Polycystin 2 are shown in lower panel (C and D). The expression of NC group of each experiment is set as 1. n = 3 independent cultures for each group.

In (A), data are presented as mean ± SD. $^{*}P < 0.05$, $^{**}P < 0.01$, $^{***}P < 0.001$ for statistical comparisons between day 0 and other time-points (ANOVA plus Bonferroni's post hoc test). In (C and D), data are presented as mean ± SD. $^{*}P < 0.05$, $^{**}P < 0.01$, $^{***}P < 0.001$ for statistical comparisons between control groups and experimental groups (Paired two-tailed *t*-test).

OE: overexpression; KnD: Knockdown; KD: nephron progenitor differentiation, NC: non-targeting control.

S7 Figure. Full length blots of FOXG1, PAX6, SIX2, and Polycystin proteins in hPSC-derived cells.

(A) Western blotting results showing expression of Polycystin 1 and Polycystin 2 in KD 6 cells derived from hESCs (H1) upon transfection of *miR-372-3p* inhibitors (n = 3 independent cultures for each group).

(B) Western blotting results showing expression of Polycystin 1 and Polycystin 2 in KD 14 cells derived from H1 upon transfection of *miR-372-3p* mimics (n = 3 independent cultures for each group).

(C) Western blotting results showing expression of Polycystin 1 and Polycystin 2 in KD 6 cells derived from iBC 1.2 upon transfection of *miR-372-3p* inhibitors (n = 3 independent cultures for each group).

(D) Western blotting results showing expression of Polycystin 1 and Polycystin 2 in KD 14 cells derived from iBC 1.2 upon transfection of *miR-372-3p* mimics (n = 3 independent cultures for each group).

Figure S1

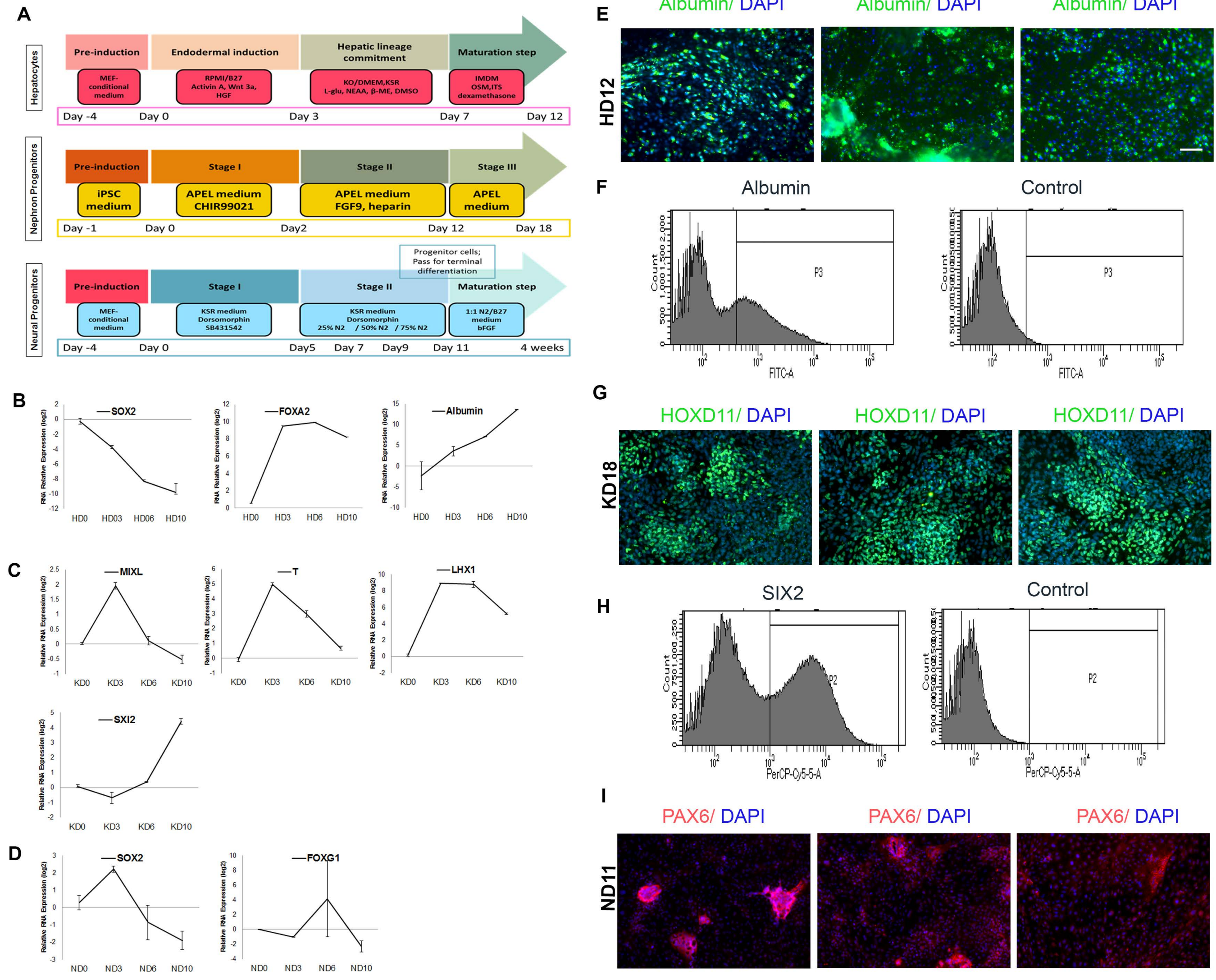

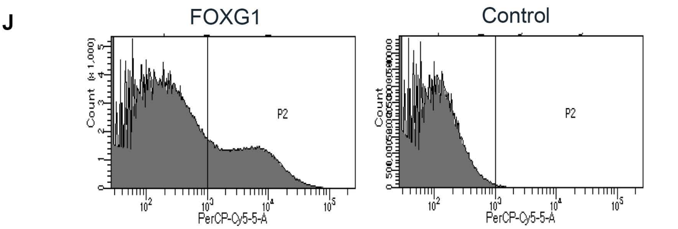

K

	% Albumin+ (IFC)	% Albumin+ (FACS)
HD 12-Cells	60.2 ± 18.7	47.8 ± 2.2

	% HOXD11+(IFC)	% SIX2+ (FACS)
KD 18-Cells	69.6 ± 8.1	43.9 ± 10.4

	% PAX6+ (IFC)	% FOXG1+ (FACS)
ND 11-Cells	53.3 ± 10.3	38 ± 2.0

Figure S2

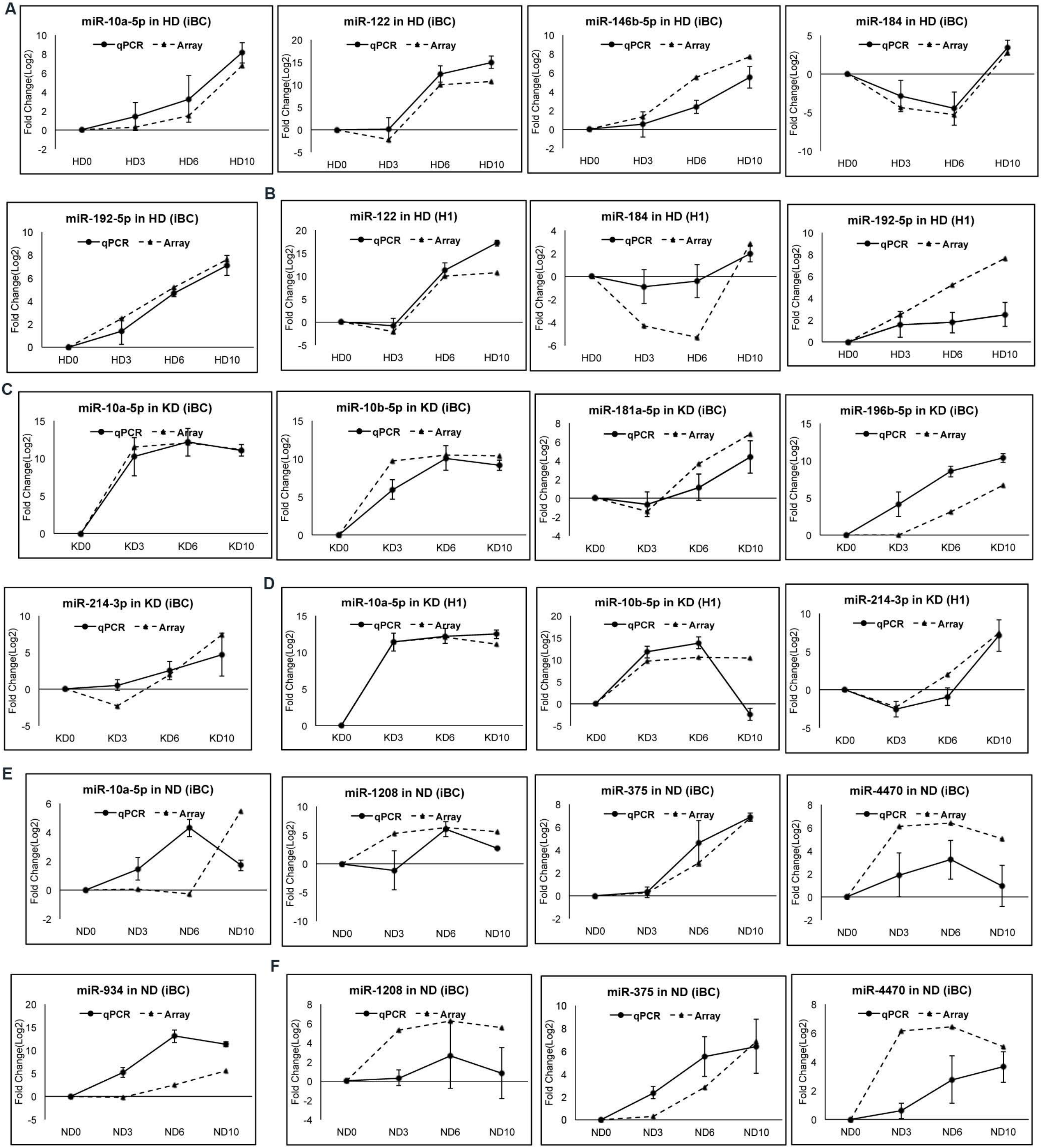
A
miR-10a-5p in HD (iBC)
miR-122 in HD (iBC)
miR-146b-5p in HD (iBC)
miR-184 in HD (iBC)
miR-192-5p in HD (iBC)
B
miR-122 in HD (H1)
miR-184 in HD (H1)
miR-192-5p in HD (H1)
C
miR-10a-5p in KD (iBC)
miR-10b-5p in KD (iBC)
miR-181a-5p in KD (iBC)
miR-196b-5p in KD (iBC)
miR-214-3p in KD (iBC)
D
miR-10a-5p in KD (H1)
miR-10b-5p in KD (H1)
miR-214-3p in KD (H1)
E
miR-10a-5p in ND (iBC)
miR-1208 in ND (iBC)
miR-375 in ND (iBC)
miR-4470 in ND (iBC)
miR-934 in ND (iBC)
F
miR-1208 in ND (iBC)
miR-375 in ND (iBC)
miR-4470 in ND (iBC)
qPCR
Array
Fold Change(Log2)
HD0
HD3
HD6
HD10
KD0
KD3
KD6
KD10
ND0
ND3
ND6
ND10

Figure S3

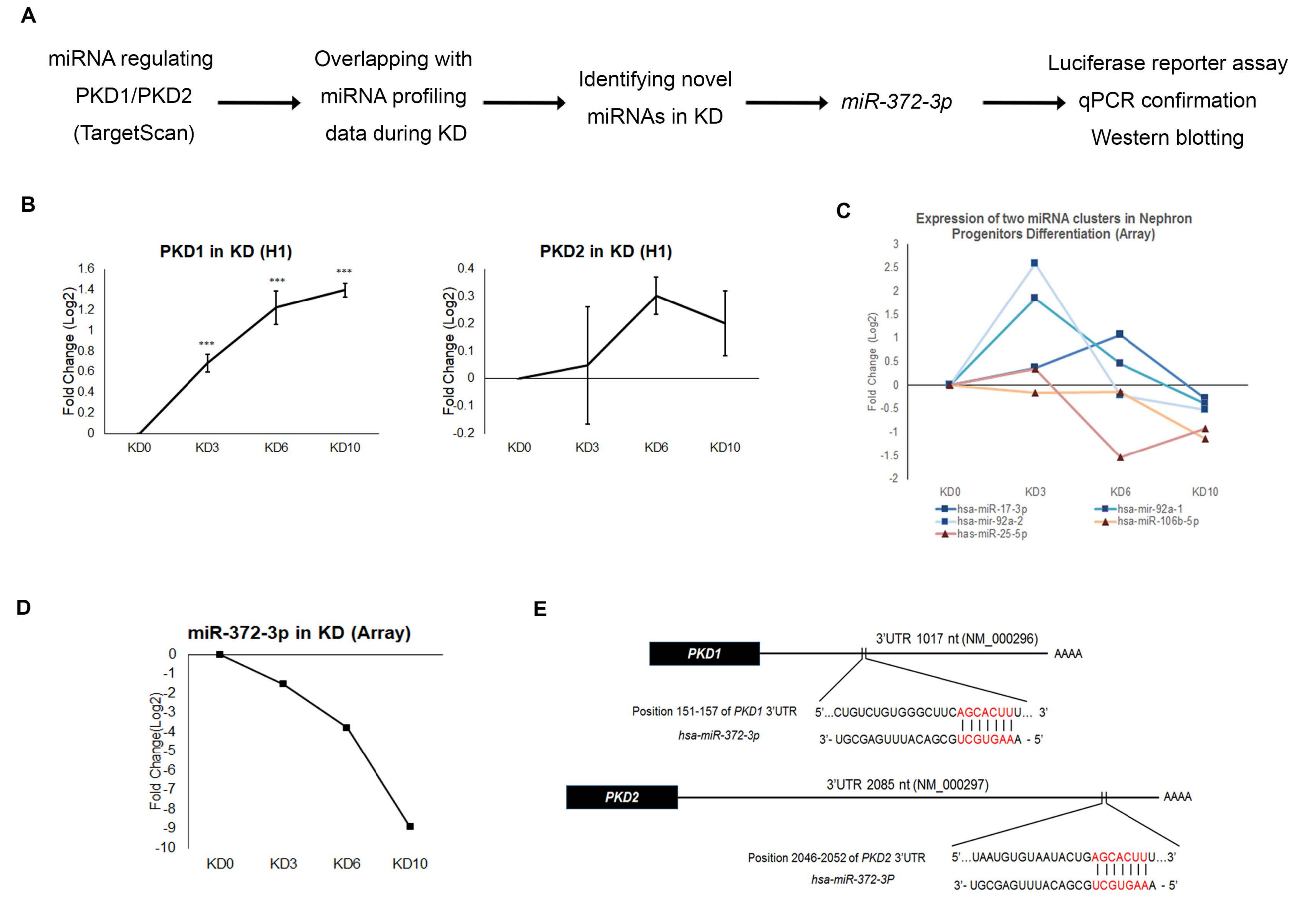
A
miRNA regulating PKD1/PKD2 (TargetScan)
Overlapping with miRNA profiling data during KD
Identifying novel miRNAs in KD
miR-372-3p
Luciferase reporter assay
qPCR confirmation
Western blotting
B
PKD1 in KD (H1)
Fold Change (Log2)
KD0
KD3
KD6
KD10
***
PKD2 in KD (H1)
Fold Change (Log2)
C
Expression of two miRNA clusters in Nephron Progenitors Differentiation (Array)
Fold Change (Log2)
hsa-miR-17-3p
hsa-mir-92a-1
hsa-mir-92a-2
hsa-miR-106b-5p
has-miR-25-5p
D
miR-372-3p in KD (Array)
Fold Change(Log2)
E
PKD1
3'UTR 1017 nt (NM_000296)
AAAA
Position 151-157 of PKD1 3'UTR
hsa-miR-372-3p
5'...CUGUCUGUGGGCUUCAGCACUUU... 3'
3'- UGCGAGUUUACAGCGUCGUGAAA - 5'
PKD2
3'UTR 2085 nt (NM_000297)
AAAA
Position 2046-2052 of PKD2 3'UTR
hsa-miR-372-3P
5'...UAAUGUGUAAUACUGAGCACUUU...3'
3'- UGCGAGUUUACAGCGUCGUGAAA - 5'

Figure S4

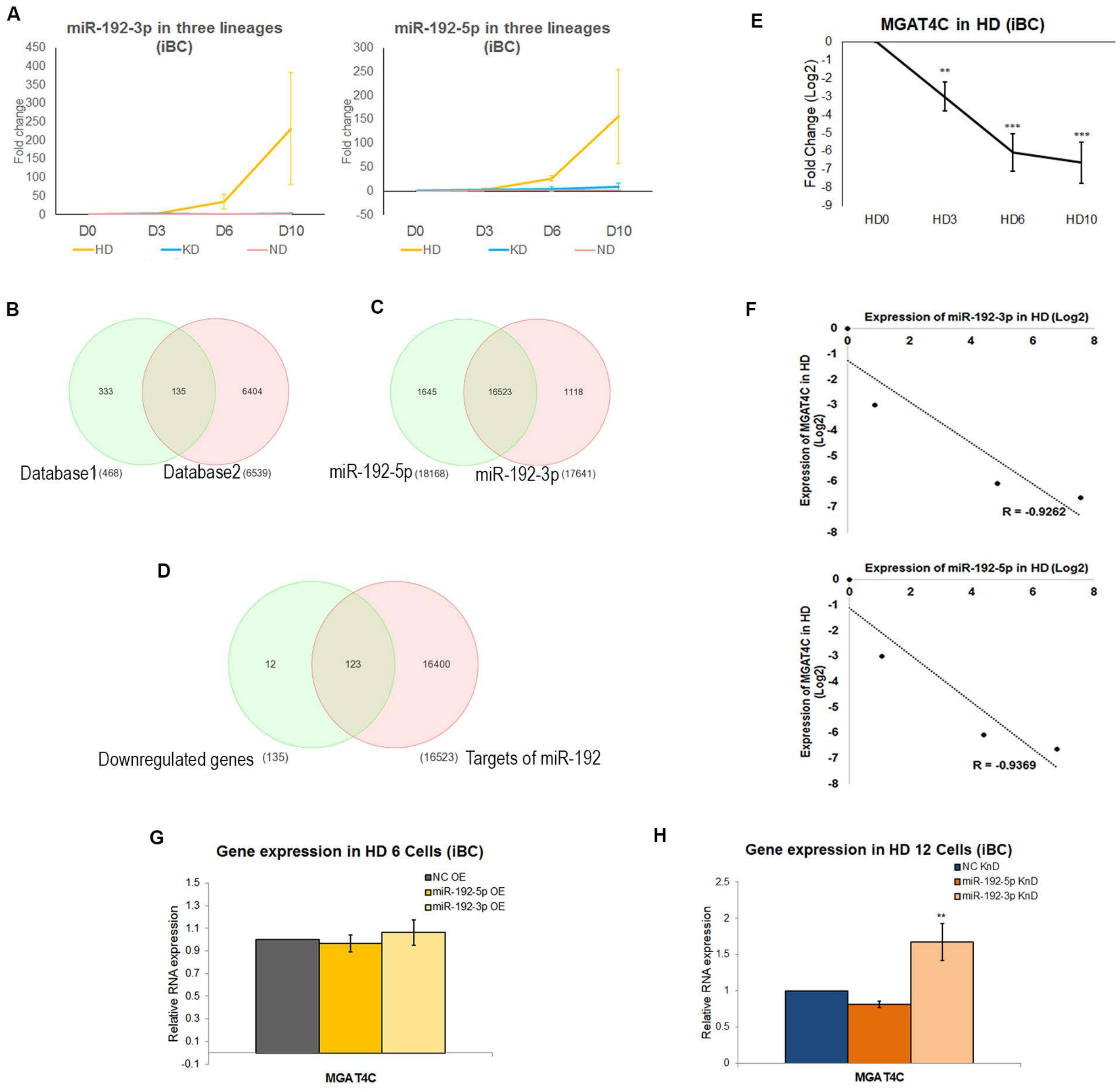
A
miR-192-3p in three lineages (iBC)
Fold change
D0
D3
D6
D10
HD
KD
ND
miR-192-5p in three lineages (iBC)
B
333
135
6404
Database1 (468)
Database2 (6539)
C
1645
16523
1118
miR-192-5p (18168)
miR-192-3p (17641)
D
12
123
16400
Downregulated genes (135)
(16523) Targets of miR-192
E
MGAT4C in HD (iBC)
Fold Change (Log2)
HD0
HD3
HD6
HD10
F
Expression of miR-192-3p in HD (Log2)
Expression of MGAT4C in HD (Log2)
R = -0.9262
Expression of miR-192-5p in HD (Log2)
R = -0.9369
G
Gene expression in HD 6 Cells (iBC)
NC OE
miR-192-5p OE
miR-192-3p OE
Relative RNA expression
MGAT4C
H
Gene expression in HD 12 Cells (iBC)
NC KnD
miR-192-5p KnD
miR-192-3p KnD

Figure S5

A

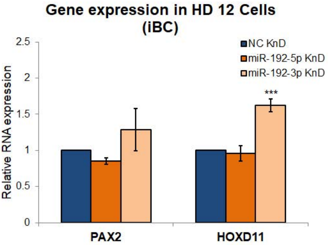

Gene expression in HD 12 Cells
(iBC)
Relative RNA expression
2.5
2
1.5
1
0.5
0
NC KnD
miR-192-5p KnD
miR-192-3p KnD
***
PAX2
HOXD11

Figure S6

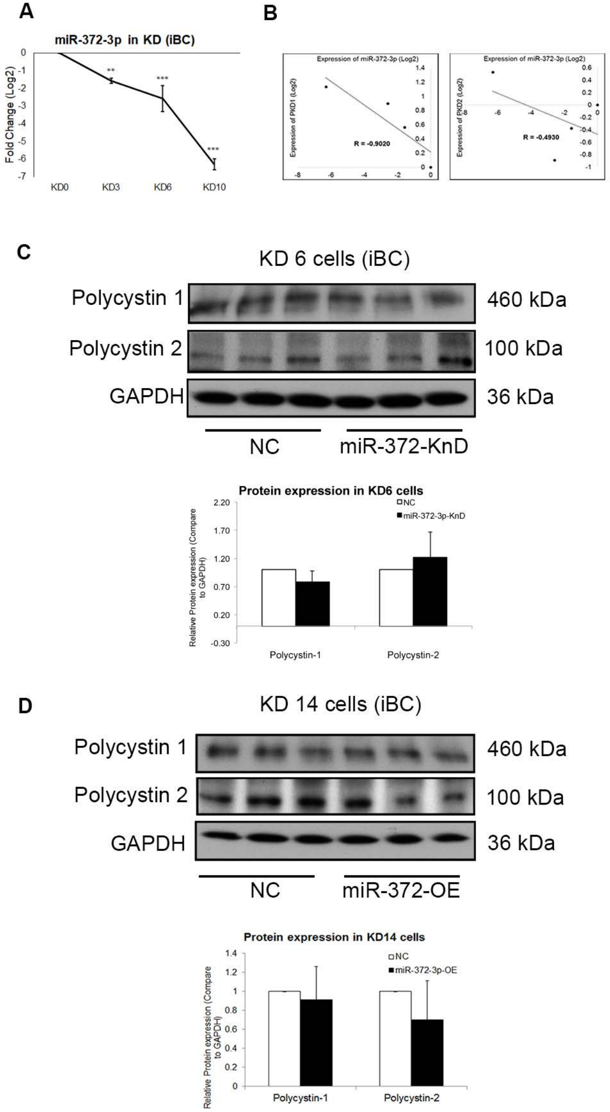
A
miR-372-3p in KD (iBC)
Fold Change (Log2)
KD0
KD3
KD6
KD10
B
Expression of miR-372-3p (Log2)
Expression of PKD1 (Log2)
R = -0.9020
Expression of PKD2 (Log2)
R = -0.4930
C
KD 6 cells (iBC)
Polycystin 1
460 kDa
Polycystin 2
100 kDa
GAPDH
36 kDa
NC
miR-372-KnD
Protein expression in KD6 cells
NC
miR-372-3p-KnD
Relative Protein expression (Compare to GAPDH)
Polycystin-1
Polycystin-2
D
KD 14 cells (iBC)
Polycystin 1
460 kDa
Polycystin 2
100 kDa
GAPDH
36 kDa
NC
miR-372-OE
Protein expression in KD14 cells
NC
miR-372-3p-OE
Relative Protein expression (Compare to GAPDH)
Polycystin-1
Polycystin-2

Figure S7

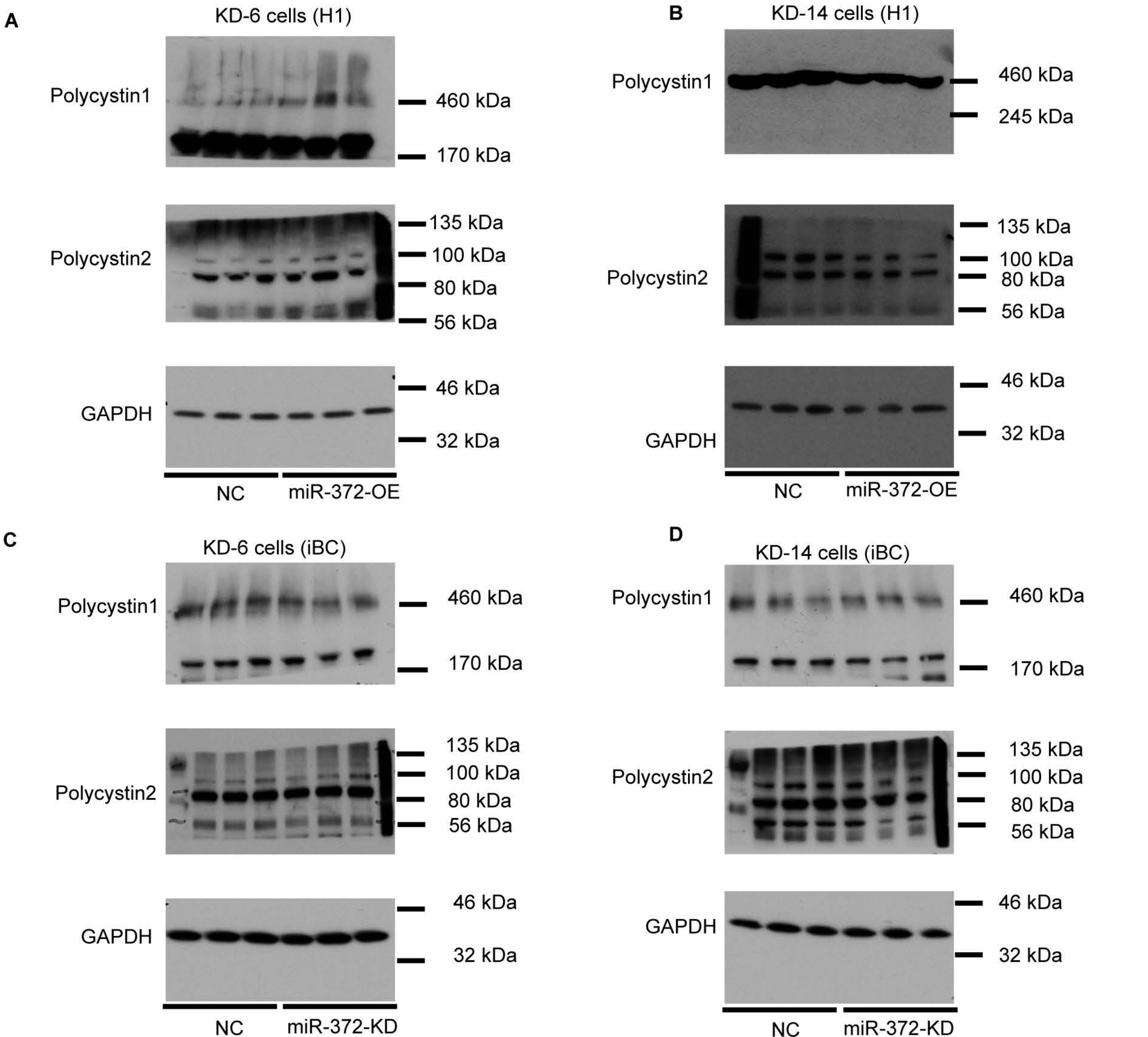
A
KD-6 cells (H1)
Polycystin1
460 kDa
170 kDa
Polycystin2
135 kDa
100 kDa
80 kDa
56 kDa
GAPDH
46 kDa
32 kDa
NC
miR-372-OE
B
KD-14 cells (H1)
Polycystin1
460 kDa
245 kDa
Polycystin2
135 kDa
100 kDa
80 kDa
56 kDa
GAPDH
46 kDa
32 kDa
NC
miR-372-OE
C
KD-6 cells (iBC)
Polycystin1
460 kDa
170 kDa
Polycystin2
135 kDa
100 kDa
80 kDa
56 kDa
GAPDH
46 kDa
32 kDa
NC
miR-372-KD
D
KD-14 cells (iBC)
Polycystin1
460 kDa
170 kDa
Polycystin2
135 kDa
100 kDa
80 kDa
56 kDa
GAPDH
46 kDa
32 kDa
NC
miR-372-KD

Supplemental Experimental Procedure

Three lineage differentiation procedures. Related to Experimental Procedure.

For hepatocyte differentiation (endoderm), a protocol established by Chen et al. was applied. Firstly, a confluency of ~90% undifferentiated iBC1.2 were treated with dispase and passed on new Matrigel-coated plates in a ratio of 1:3. Thereafter, cells were pre-induced with MEF conditional medium for 3-5 days until the cell density reached ~70% confluency (HD 0 cells). The pre-induced cells were then cultured in endodermal induction medium, which are RPMI 1640 medium with 1XB27 supplement (Gibco, A14867-01) and 100 ng/ml Activin A (R&D Systems, 338-AC) and 10 ng/ml HGF (R&D Systems, 294-HGN) and 50 ng/ml Wnt 3a (R&D Systems, 5036-WN), for 3 days. Then the medium was replaced by hepatic lineage commitment medium (Knockout DMEM medium with 20% knockout serum replacement and 1X GlutaMAX and 1X MEM Non-Essential Amino Acids and beta-mercaptoethanol and 1% DMSO) for 4 days, and finally changed to maturation medium, which are IMDM medium with 20ng/ml Oncostatin M (Gibco, PHC5015) and 0.5 μM dexamethasone (Sigma, D4902-25MG) and 500mg/ml ITS premix (Corning, 354351). Media was changed every day.

For nephron progenitor differentiation, a protocol established by Takasato et al. was employed. Firstly, iBC1.2 were individualized with Accutase (StemPro, A11105001) and plated onto new Matrigel-coated plates at a density of 1X104 cell/cm2. After one-day pre-induction with MEF conditional medium (KD 0 cells), the cells were cultured in Stage I medium, which are STEMdiff APEL medium (StemCell Technologies, 05210) with 8 μM CHIR99021 (Selleck Chemicals, S-2924) for 2 days. Thereby, the cells were exposed to Stage II medium, which are APEL medium with 200 ng/ml FGF9 (ThermoFisher, PHG0194) and 1 μg/ml heparin. After another 12 days, the cells were cultured in plain APEL medium. At the 6th day of induction, the cells were subcultured to new Matrigel-coated plates in the ratio of 1:6. The media were changed every other day.

For neural progenitor differentiation, an established protocol modified by Mohamad et al. were used. Similar to pre-treatment of nephron progenitor induction, the single hiPSCs were seeded in a density around 1.95X 104 cell/cm2 with MEF conditional medium (KD 0 cells). After the cells reached ~80% confluency, the medium was replaced with KSR medium (KO DMEM medium with 15% KSR and 1X Glutamax

and 1X MEM NEAA and beta-mercaptoethanol) and 3 μM Dorsomorphin (Sigma Aldrich, P5499-5M) and 10 μM SB431542 (EMD Millipore, 616464) for the first 5 days. Starting from day 5, the cells were culture in a combination of KSR medium and N2 medium (DMEM/F12 medium with 1X N2 supplement and 1X GlutaMAX) with Dorsomorphin. The ratio of KSR medium and N2 medium was shift from 4:1, 1:1, to 1:4 at day 5, day 7, and day 9, respectively. The media was changed every day. For further differentiation into neuron, neural progenitors were individualized with accutase and filtered with 20 μm nylon net (EMD Millipore, SCNY00020) and seeded on fresh Matrigel-coated plate. The cells were exposed to a combination of N2 medium and B27 medium (Neurobasal medium with 1X B27 supplement and 1X GlutaMAX and 1X MEM NEAA) with 10 ng/ml bFGF for another 4 weeks. The media were changed every third days.

Flow cytometry

A vector containing Albumin promoter sequence fused to a GFP reporter gene was transfected into HD 6 cells. HD 12 cells, KD 18 cells, and ND 11 cells were harvested by centrifugation at 400 × g at 4°C for 10 min. Permeabilization was done for KD 18 cells and ND 11 cells on ice for 30 min with Fix Buffer I (557870, BD Biosciences). KD 18 cells and ND 11 cells were stained in Stain buffer (554657, BD Pharmingen) with the SIX2 antibody and FOGX1 antibody, respectively, for 40 min. HD 12 cells, KD 18 cells and ND 11 cells were then washed and measured on the BD LSR Fortessa and data analysis was done using FACSDiva software and Microsoft Excel.

Supplemental Antibody

Target	Supplier	Product Code	Dilution Ratio
OCT4	Millipore	MAB4401	1:100
Albumin	Abcam	Ab10241	1:500
PAX2	Zymed Laboratories	71-6000	1:200
SIX2	Proteintech	11562-1-AP	1:200
HOXD11	Sigma-Aldrich	SAB1403944	1:200
Jagged1	Abcam	Ab7771	1:200
WT1	Santa Cruz Biotechnology	sc-192	1:100
CDH6	Sigma-Aldrich	HPA007047	1:100
ECAD	BD Biosciences	610182	1:200
PAX-6	Covance	PRB-278P	1:100
FOXG1	Abcam	Ab18259	1:10
TUJ1	Covance	A488-435L	1:500
Synapsin I	Millipore	AB1543	1:400
Nestin	Millipore	MAB5326C3	1:100
NeuN	Millipore	MAB377C3	1:100
Neurofilament	Millipore	MAB5256X	1:100
SOX17	Abcam	Ab84990	1:50
SOX1	R&D System	AF3369	1:50
Polycystin 1	Santa Cruz Biotechnology	sc-130554	1:50
Polycystin 2	Santa Cruz Biotechnology	25749	1:200
